# Spatiotemporal decoding of skin biology: development, aging, disease, and regeneration

**DOI:** 10.1093/burnst/tkag019

**Published:** 2026-02-28

**Authors:** Xinran Ding, Runzhi Huang, Yixu Li, Zhaofan Xia, Shizhao Ji

**Affiliations:** Department of Burn Surgery, The First Affiliated Hospital of Naval Medical University, Shanghai, 200433, China; Research Unit of Key Techniques for Treatment of Burns and Combined Burns and Trauma Injury, Chinese Academy of Medical Sciences, Shanghai, 200433, China; Department of Burn Surgery, The First Affiliated Hospital of Naval Medical University, Shanghai, 200433, China; Research Unit of Key Techniques for Treatment of Burns and Combined Burns and Trauma Injury, Chinese Academy of Medical Sciences, Shanghai, 200433, China; Department of Burn Surgery, The First Affiliated Hospital of Naval Medical University, Shanghai, 200433, China; Research Unit of Key Techniques for Treatment of Burns and Combined Burns and Trauma Injury, Chinese Academy of Medical Sciences, Shanghai, 200433, China; Department of Burn Surgery, The First Affiliated Hospital of Naval Medical University, Shanghai, 200433, China; Research Unit of Key Techniques for Treatment of Burns and Combined Burns and Trauma Injury, Chinese Academy of Medical Sciences, Shanghai, 200433, China; Department of Burn Surgery, The First Affiliated Hospital of Naval Medical University, Shanghai, 200433, China; Research Unit of Key Techniques for Treatment of Burns and Combined Burns and Trauma Injury, Chinese Academy of Medical Sciences, Shanghai, 200433, China

**Keywords:** Spatiotemporal omics, Skin biology, Single-cell sequencing, Spatial transcriptomics, Regenerative medicine, Aging, Skin disease

## Abstract

Skin shows distinct temporal dynamics and spatial heterogeneity during development, aging, disease, and regeneration. Although single-cell sequencing has revealed cellular diversity, its lack of spatial context limits the ability to characterize cells within their native tissue microenvironment. Factors such as acute injury and chronic wounds spatiotemporally disrupt skin homeostasis and induce complex remodeling and functional changes. Understanding these dynamic processes with spatiotemporal resolution remains a challenge in skin biology. Recent advances in spatiotemporal omics make it possible to integrate single-cell sequencing, spatial omics, and time series analyses, allowing the preservation of *in situ* cellular positions and revealing gene expression dynamics and intercellular networks. These technologies have reshaped the understanding of skin development and wound healing and have promoted advances in precision medicine and regenerative therapies. In this review, the applications of, recent advances in, and clinical translation potential of spatiotemporal omics in skin research are summarized. The construction of a high-resolution, spatiotemporal cellular atlas across the human skin life cycle will help identify key biomarkers, optimize regenerative strategies, and support personalized therapies.

## Highlights

- Multidimensional integration: spatiotemporal omics unifies single-cell profiling, spatial localization, temporal resolution, and multiomics analyses to preserve the *in situ* context, allowing the resolution of lineage trajectories and intercellular communication networks from the molecular to the tissue scale.
- Redefining cutaneous biology: spatiotemporal omics has revolutionized the understanding of skin physiology, enabling high-dimensional reconstruction of cellular heterogeneity and tissue organization across the continua of development, aging, disease, and regeneration.
- Translational impact: by linking fundamental mechanistic insights to clinical application, spatiotemporal omics allows the identification of novel therapeutic targets, paving the way for next-generation regenerative strategies and precision dermatology.

## Background

Skin is a complex organ composed of diverse cell types and multiple layers, including the epidermis, dermis, and subcutaneous tissue. It contains keratinocytes, fibroblasts, melanocytes, and immune cells, as well as specialized structures such as hair follicles (HFs), vasculature, and nerves. These cells exhibit an ordered spatial arrangement with extracellular matrix (ECM) components, maintaining tissue homeostasis and functional coordination through mutual interactions [1–3]. This anatomical ‘niche’ of skin not only has a defined layered architecture but also undergoes complex, multiscale, and multistate dynamics during physiological and pathological processes.

**Figure 1:**
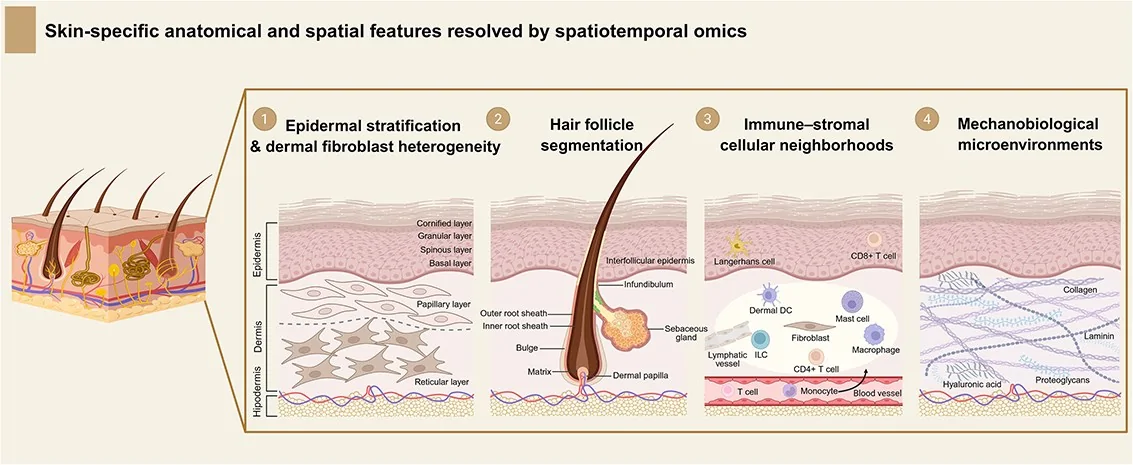
Skin-specific anatomical and spatial features resolved by spatiotemporal omics. Schematic illustration of the full-thickness skin architecture and representative spatial features captured by spatiotemporal omics, namely, epidermal stratification and dermal fibroblast heterogeneity, hair follicle segmentation, immune–stromal cellular neighborhoods, and mechanobiological microenvironments defined by extracellular matrix organization

From embryonic development to aging and from disease onset to tissue repair, skin undergoes diverse biological processes across life stages. During development, stem cells of distinct lineages differentiate into various skin cell types and contribute to the formation of appendages such as HFs and glands [4]. In adulthood, the skin maintains homeostasis and performs functions such as sensory perception and immune defense [5]. With aging, it exhibits characteristic hallmarks, namely, structural disorganization, barrier dysfunction, and the accumulation of proinflammatory factors [6]. Upon injury, skin-resident cells rapidly undergo reprogramming to enable wound closure and functional restoration [7, 8]. In contrast, pathological conditions, such as psoriasis, vitiligo, and keloids, frequently disrupt cell–cell communication (CCC), migratory behavior, and activation/inhibition dynamics, leading to aberrant cellular community structures and impaired tissue remodeling [9, 10]. These biological events occur within highly structured and spatially constrained tissue, underscoring the need for analytical approaches that capture cellular dynamics while preserving spatial context (Figure 1).

Spatial biology is entering a transformative era. Early skin research relied on genetic lineage tracing, flow cytometry, and transcriptome microarrays; however, these methods lacked the ability to capture both spatial information and cellular heterogeneity. Spatiotemporal omics is a high-throughput technology that integrates spatial profiling, single-cell sequencing, and temporal analysis. By retaining the spatial architecture and temporal dynamics of cells, spatiotemporal omics enables a comprehensive characterization of the transcriptomic, proteomic, and metabolomic landscapes. Unlike conventional single-cell or bulk omics approaches that provide only static snapshots at a single time point, spatiotemporal omics highlights dynamic changes and offers unique advantages for gaining mechanistic insights [11]. To comprehensively evaluate the current landscape and future directions of skin spatiotemporal omics, we systematically searched the Web of Science Core Collection (WOSCC) for relevant studies up to 5 January 2026. The search strategy was based on the following terms: TS = (‘spatiotemporal omics’ OR ‘spatial transcriptomics’ OR ‘single-cell sequencing’ OR ‘scRNA-seq’ OR ‘multi-omics’ OR ‘skin spatial’ OR ‘skin transcriptomics’ OR ‘spatiotemporal transcriptomics’) AND TS = (skin OR cutaneous OR wound OR scar OR burn OR ulcer OR keloid OR psoriasis OR dermatitis OR eczema OR ‘skin aging’ OR ‘skin regeneration’). A total of 3113 publications were retrieved. After the document type was limited to articles, 1516 publications were retained for downstream bibliometric analysis, which was subsequently performed using the Bibliometrix R package [12].

Analysis of the field’s trajectory indicated that progress has been driven by several landmark advances. In 2016, Joost *et al.* pioneered the systematic application of single-cell RNA sequencing (scRNA-seq) to mouse skin, meticulously constructing a molecular atlas comprising 25 distinct cell populations and their associated differentiation trajectories [13]. In subsequent studies, these insights were extended to human skin, revealing the transcriptional heterogeneity of the epidermis [14]. Given the highly ordered, stratified architecture of the skin, early investigative efforts sought to resolve the spatial distribution of dermal fibroblast (dFB) subpopulations through physical microdissection combined with the Smart-seq2 method, achieving a preliminary spatial delineation of tissue heterogeneity [15]. The advent of next-generation spatiotemporal omics technologies has provided a systematic solution for capturing high-content molecular information while preserving the native tissue architecture. In 2020, spatial transcriptomics (ST) was successfully applied to intact skin tissue sections, allowing the collection of on-slide, patch-scale transcriptomic measurements in disease models such as cutaneous squamous cell carcinoma (cSCC) [16]. This technological breakthrough furnished a critical tool for the systematic investigation of skin development, disease pathogenesis, and microenvironmental interactions and, to some extent, marked the formal entry of skin biology into a spatiotemporal omics-driven research phase. Building upon these trends, this review provides a summary of recent technological advances and their applications in skin development, aging, disease, and regeneration while highlighting prospects for clinical translation.

## Review

### Overview of spatiotemporal omics in skin biology

**Figure 2:**
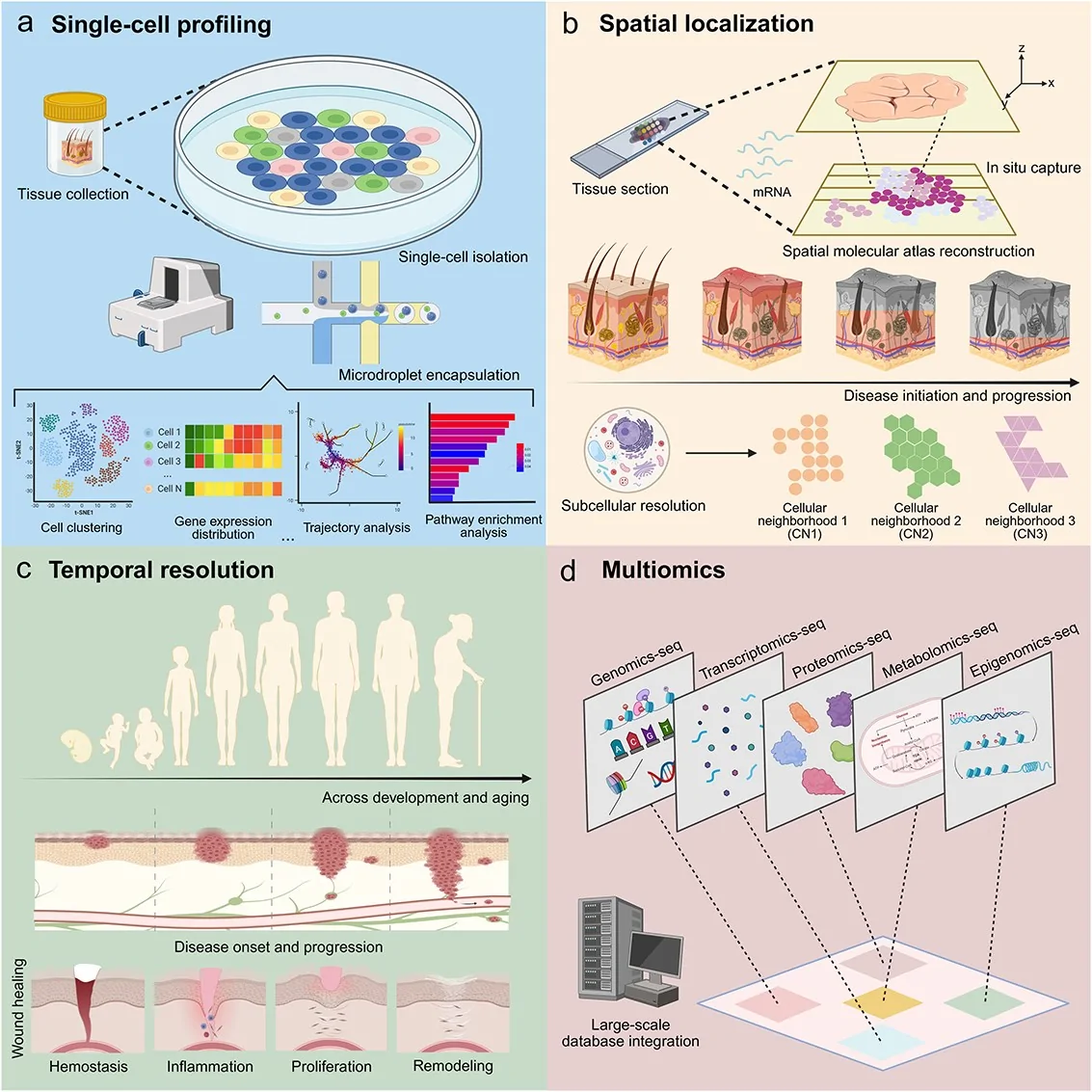
Overview of spatiotemporal omics for the study of skin biology. Spatiotemporal omics integrates four complementary dimensions to map the cellular and molecular landscapes of skin. (**a**) Single-cell profiling: scRNA-seq enables high-throughput transcriptional analysis at single-cell resolution, revealing cell heterogeneity, lineage trajectories, and functional states. (**b**) Spatial localization: spatial omics preserves tissue architecture and allows the reconstruction of molecular atlases at cellular to subcellular resolution, capturing microenvironmental organization and disease progression. (**c**) Temporal resolution: multi-time point sampling and lineage tracing approaches allow dynamic tracking of stem cell fate, wound healing, and age-associated remodeling across the lifespan. (**d**) Multiomics: combining genomic, transcriptomic, proteomic, metabolomic, and epigenomic layers provides systems-level insights into skin homeostasis, inflammation, and regeneration

Skin spatiotemporal omics allows the resolution of information on multiple scales, from single-cell molecular profiles to dynamic functional systems. This approach involves four main dimensions (Figure 2): (i) single-cell profiling (isolating gene expression information for each cell type); (ii) spatial localization (identifying the cellular distribution within tissues and the interactions among cells); (iii) temporal resolution (tracing cell state transitions and lineage trajectories over time); and (iv) multiomics (combining analyses of deoxyribonucleic acid (DNA) sequences, regulatory elements, posttranscriptional modifications, protein functions, and metabolic pathways).

#### Bibliometric analysis

A total of 1516 articles on skin spatiotemporal omics were indexed in WOSCC up to 5 January 2026. The data presented in Figure S1 demonstrate consistent and significant increases in the numbers of annual publications and total citations, with explosive growth observed in recent years (2019–2025). In terms of the country/region distribution, the United States, China, and Germany are the top three contributors to the field (Figure S2a). The proportions of single-country publications (SCP) and multiple-country publications (MCP) indicate that high-level international collaboration is a major driving force for technological breakthroughs (Figure S2b), highlighting the highly interdisciplinary nature of this rapidly evolving research frontier. Source analysis based on local citations (Figure S3a) further identified Nature Communications as one of the most influential academic journals in this field, with the highest local citation frequency (*n* = 2434).

**Figure 3:**
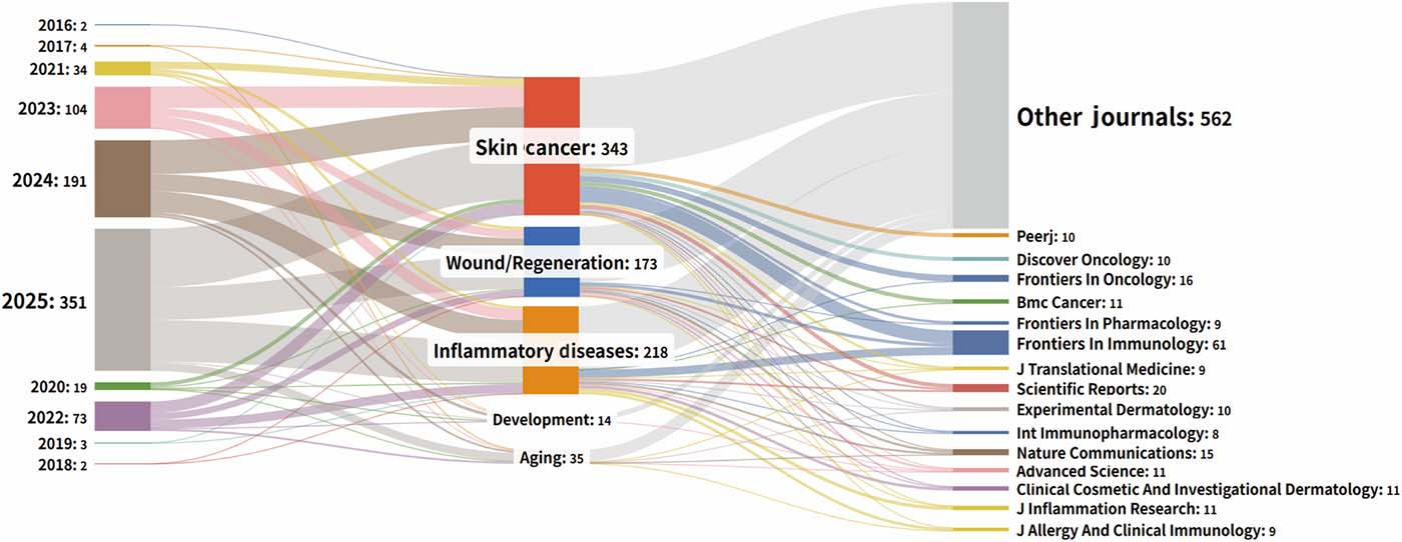
Sankey diagram linking the publication year, major research themes, and journal sources of skin spatiotemporal omics studies. The left column shows the annual scientific production (number of articles per year). The middle column summarizes the main research themes and their total outputs (skin cancer, *n* = 343; inflammatory diseases, *n* = 218; wound/regeneration, *n* = 173; aging, *n* = 35; development, *n* = 14). The right column lists the most productive journals and ‘other journals’ (*n* = 562). The width of each link is proportional to the number of articles connecting a given year to a theme and a theme to a journal, illustrating the temporal evolution of topic emphasis and the distribution of publication venues. The Sankey diagram was generated using SankeyMATIC (https://sankeymatic.com/)

The Sankey diagram (Figure 3) shows the multidimensional links among research themes, temporal evolution, and journal sources. From 2016–2020, the overall publication output was limited, and studies focused predominantly on application-oriented contexts such as skin cancer, wound healing, and regeneration. Since 2021, the breadth of research hotspots has expanded rapidly to include inflammatory diseases, aging, and development, reflecting a transition from analyses of single pathological contexts toward a broader decoding of skin dynamics across the lifespan. With respect to the journal distribution, Frontiers in Immunology (*n =* 61), Scientific Reports (*n =* 20), Frontiers in Oncology (*n =* 16), and Nature Communications (*n =* 15) were identified as highly productive sources, underscoring the strong multidisciplinary integration and translational potential of the field. Notably, the high-impact document analysis (Figure S3b and c) revealed that the top-ranked paper under both global and local citation metrics was the CellChat methodology article [17], indicating that the field relies strongly on its CCC inference and network analysis framework as a key computational foundation.

The high-frequency keyword and thematic map analysis further delineated the core intellectual structure of the field (Figure S4). Themes with both high centrality and high density, including ‘skin,’ ‘cells,’ and ‘single-cell RNA sequencing,’ clustered in the motor themes quadrant, representing current research hubs; in contrast, ‘cancer,’ ‘melanoma,’ and ‘immunotherapy’ were classified as emerging themes or declining themes, suggesting their identity as important future directions. Moreover, ‘expression,’ ‘multi-omics,’ and ‘gene’ were increasingly consolidated into basic themes, reflecting their foundational role in the knowledge base. Collectively, these findings indicate that skin spatiotemporal omics is shifting from a focus on localized tissue characterization toward a life cycle-scale, dynamic framework, spanning four major biological contexts, namely, development, aging, disease, and regeneration, with this observation providing a quantitative foundation for the subsequent sections of this review.

#### Technological and algorithmic evolution

The emergence of scRNA-seq in 2009 provided an essential foundation for spatial omics and marked the first milestone in spatiotemporal omics [18]. Through microdroplet encapsulation, microwell capture, and barcoded library construction, scRNA-seq enables high-throughput transcriptome profiling, greatly expanding insights into skin heterogeneity, cell lineages, and cell functional states. Current mainstream platforms, including the 10x Genomics [19], Drop-seq [20], Smart-seq2 [21], and CEL-seq2 [22] platforms, offer distinct advantages in terms of throughput, cost, and transcript coverage and have been widely applied in skin research. Building upon single-cell isolation techniques, spatial omics technologies have enabled the precise reconstruction of three-dimensional (3D) tissue architecture and are categorized primarily into imaging-based and sequencing-based approaches [11]. Early techniques relied on manual dissection or positional sampling for regional sequencing. Optical tissue clearing (OTC) is a critical sample engineering strategy in skin research that reduces light scattering caused by dermal collagen and other ECM components, thereby markedly increasing the imaging depth and signal-to-noise ratio in thick tissues [23–25]. Because of these features, imaging-based spatial omics techniques (e.g. MERFISH/MERSCOPE [26], HCR-FISH [27], and CODEX/CyCIF [28, 29]) can be used to construct high-resolution 3D molecular maps *in situ*. Sequencing-based spatial omics approaches are driving measurements toward high-throughput and near-whole-transcriptome coverage. Introduced in 2016, ST was the pioneering method to use barcoded oligonucleotide probes on slides to capture mRNA *in situ*, enabling large-scale spatial molecular mapping [30]. Subsequent technologies such as 10× Genomics Visium [31], Slide-seq [32], HDST [33], and Stereo-seq [34] have provided further increases in resolution and throughput, being used to successfully resolve the microenvironmental distribution of multilayered skin structures and functional units (e.g. HFs, sweat glands, vasculature, and immune niches). More recently, Pixel-seq and high-resolution Stereo-seq have achieved subcellular resolution, enabling studies of microscale structural remodeling and cellular neighborhood interactions [35, 36]. Given the dynamic nature of skin biology, incorporating temporal information is essential for understanding its physiological and pathological processes. Multi-time point sampling designs [37], often combined with lineage tracing, enable the analysis of dynamic changes in skin tissues within a spatial context. RNA velocity models [38], which infer the direction and rate of cell state transitions on the basis of the relative abundances of spliced and unspliced mRNA, together with pseudotime algorithms, further allow the reconstruction of cell fate trajectories and tissue dynamics. Furthermore, incorporating multiple omics layers is an emerging trend. The integration of transcriptional, proteomic, metabolic, and epigenetic information within spatial contexts is enabled by platforms such as spatial CITE-seq [39], which coindexes whole transcriptomes with high-plex antibody-derived protein tags *in situ*; spatial ATAC-seq [40], which maps spatially resolved chromatin accessibility in tissue sections; and scSpaMet [41], which couples untargeted spatial metabolomics with targeted multiplex protein imaging in the same tissue. These advances are driving skin research and clinical applications toward a systems-level, multidimensional paradigm.

The massive datasets generated by spatiotemporal omics approaches necessitate robust data preprocessing, integration, and biological interpretation. To address these challenges, specialized algorithms have been developed for tasks such as spatial alignment, niche reconstruction, spatial partitioning, CCC, and dynamic trajectory inference (the principles and performance of these algorithms are summarized in Supplementary Table S1). From quality control and normalization of raw data to cell type annotation, spatial mapping, signaling inference, trajectory reconstruction, and fate prediction, every analytical step relies heavily on the selection of appropriate algorithms and parameters. The accuracy of these analyses determines the reliability of the related biological discoveries. On the basis of the above platforms and algorithms, we also compiled currently available large-scale spatiotemporal omics datasets of skin, which represent valuable resources for skin biology research (Table 1) [42–53].

**Table 1:** Summary of available skin single-cell and spatial omics atlases and databases

Database	Date	Species	Data type	Link	Gene query/Visualization support	Key information	Ref.
The Human Skin Atlas	2020	Human (healthy skin)	Spatial proteomics	https://skin.science/	Yes	Quantitative spatial and cell type-resolved proteomic map of healthy human skin, revealing structural and immune protein distributions across layers	[42]
SpatialDB	2020	Human, mouse	ST	http://spatialomics.org/SpatialDB/index.php	Yes	Published ST datasets of human and mouse skin, enabling the exploration of layer-specific expression patterns and spatially variable genes	[43]
HCA Skin Network	2021	Human (7–10 PCW)	scRNA-seq	https://developmental.cellatlas.io/fetal-skin	Yes	Collection of over 500 000 single cells from fetal (7–10 PCW), healthy adult, and diseased (AD, psoriasis) skin, enabling comparisons of developmental, healthy, and inflammatory states	[44]
Embryonic Mouse Skin Portal	2023	Mouse (embryo)	scRNA-seq + ST	http://kasperlab.org/tools	Yes	Fibroblast lineage differentiation trajectories in mouse skin from the embryonic to adult stages, driving HF, muscle, and fascia formation	[13, 45, 46]
HMS Browser	2023	Human, mouse	scRNA-seq + ST	https://rstudio-connect.hpc.mssm.edu/hmsbrowser/	Yes	Human–mouse cross-species comparisons of cell type-specific gene expression, providing annotations for cells from the IFE, pilosebaceous unit, and mesenchymal compartments	[47]
Skin Cancer Atlas Browser	2023	Human	scRNA-seq + ST	https://skincanceratlas.com/	Yes	Single-cell atlas of SCC, BCC, melanoma, and healthy skin containing over 50 000 cells, enabling spatial mapping of rare immune cells and cross-cancer interaction networks	[48]
STOmicsDB	2023	Multiple	ST	https://db.cngb.org/stomics/	Yes	Curated ST datasets of human and mouse skin (healthy and diseased), enabling gene-level spatial queries and visualization of skin cell types, microenvironmental organization, and cell–cell interactions	[49]
WebAtlas (HCA Dev Cell Atlas)	2024	Human (7–17 PCW)	scRNA-seq + ST	https://developmental.cellatlas.io/fetal-skin	Yes	Developmental map of immune maturation and morphogenesis in fetal skin (7–17 PCW), defining key stages of skin formation	[50]
Spatial Skin Atlas	2024	Human	scRNA-seq + ST + ISS	https://spatial-skin-atlas.cellgeni.sanger.ac.uk/	Yes	Spatial atlas of healthy adult skin and BCC across anatomical sites	[51]
Space Omics and Medical Atlas	2024	Human (astronaut skin, before and after spaceflight)	scRNA-seq + ST + scATAC-seq + Metagenomics/metatranscriptomics	https://soma.weill.cornell.edu/	Yes	Transcriptomic profiles of skin under microgravity conditions, revealing altered immune responses and gene expression patterns	[52]
Pediatric Skin Atlas	2025	Human (pediatric/neonatal/childhood)	scRNA-seq + ST	https://chanzuckerberg.com/science/programs-resources/cell-science/pediatric-networks/single-cell-multi-omic-and-spatial-cell-atlas-of-pediatric-skin/	Yes	Reference atlas of pediatric skin maturation, defining milestones for understanding childhood skin biology, diseases, and differences from adult skin	[53]

Abbreviations: ST, spatial transcriptomics; scRNA-seq, single-cell RNA sequencing; PCW, postconceptional week; AD, atopic dermatitis; HF, hair follicle; IFE, interfollicular epidermis; SCC, squamous cell carcinoma; BCC, basal cell carcinoma

### Mapping development and aging: spatiotemporal landscapes across the skin lifespan

**Figure 4:**
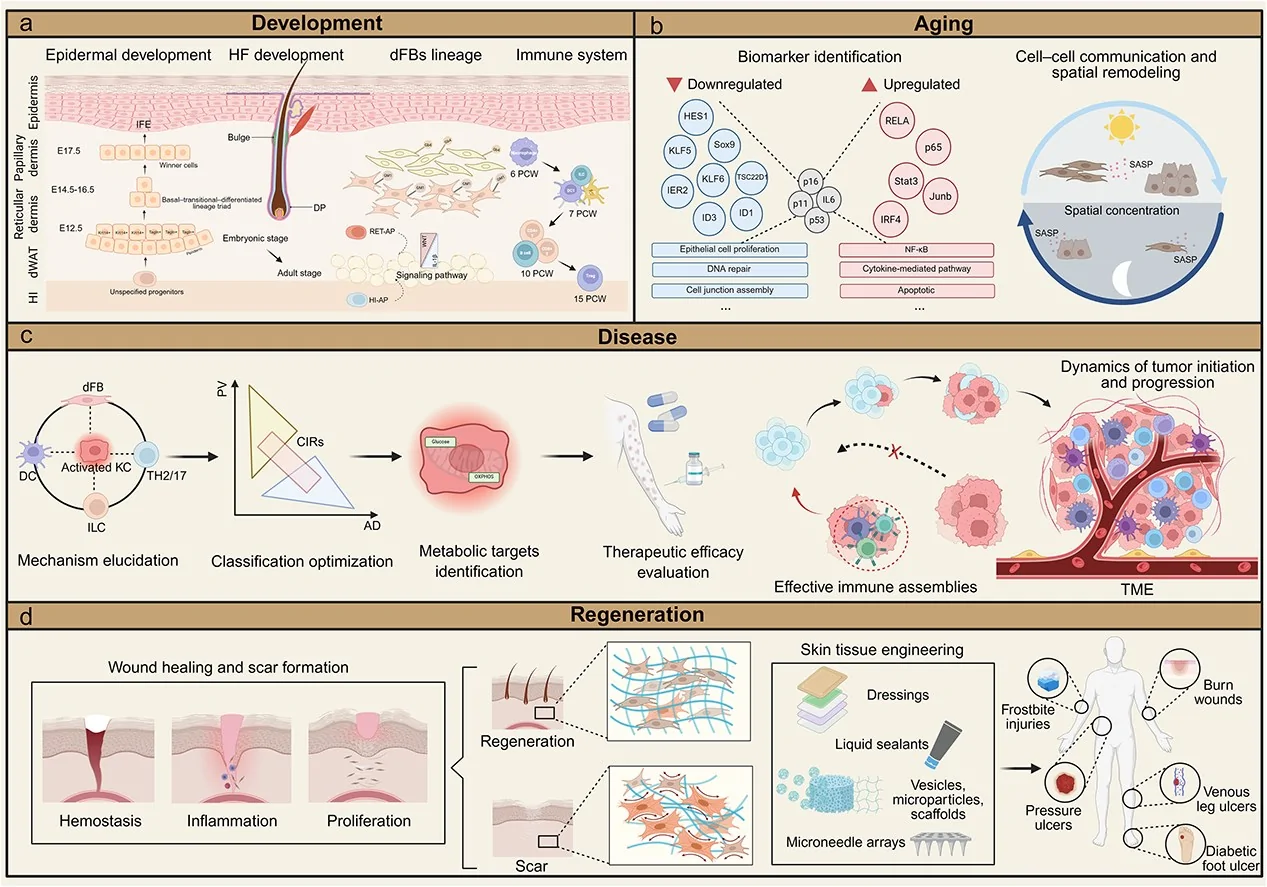
Spatiotemporal omics insights into skin development, aging, disease, and regeneration. (**a**) Development. Spatiotemporal omics enables high-resolution dissection of epidermal development [45, 59–61], HF development, lineage differentiation of dFBs [62, 63], and early immune system establishment [44, 50, 64]. Integrated transcriptomic, epigenomic, and signaling analyses reveal dynamic trajectories, hierarchical lineage specification, and niche-dependent regulation. (**b**) Aging. Identification of aging biomarkers highlights the transcriptional shift from stemness to inflammatory remodeling, with downregulation of genes related to cell proliferation and DNA repair, and upregulation of cytokine-mediated and apoptotic pathways [65, 66]. Spatial analyses uncover the role of SASP in mediating CCC, spatial concentration, and microenvironmental remodeling [6]. (**c**) Disease. Spatiotemporal omics advances mechanistic dissection of chronic inflammatory dermatoses (e.g. AD, PV), optimizes molecular classification of CIRs [67], identifies metabolic vulnerabilities [68], and evaluates therapeutic efficacy. In skin cancers, it enables mapping of immune cell assemblies, dissection of tumor-microenvironment crosstalk, and reconstruction of tumor initiation and progression dynamics within the TME. (**d**) Regeneration. Applications in wound healing and scar formation reveal cellular and molecular dynamics across hemostasis, inflammation, proliferation, and remodeling phases. Integration with tissue engineering facilitates healing by guiding biomaterial design, evaluating therapeutic responses, and elucidating mechanisms of action. Emerging platforms including vesicles, microparticles, scaffolds, and microneedle arrays [69, 70]—support translational strategies for treating burn wounds, diabetic foot ulcers, pressure ulcers, frostbite injuries, and other refractory wounds. *dFBs* dermal fibroblasts, *HI* hypodermal/interstitial, *E17.5* embryonic day 17.5, *PCW* post-conceptional week, *HF* hair follicles, *CCC* cell–cell communication, *SASP* senescence-associated secretory phenotype, *CIRs* clinical/histopathologically indeterminate rashes

Skin development is a process involving synchronized orchestration among the epidermis, dermis, appendages, and immune system [54–56]. This process involves stratified barrier formation, epithelial–mesenchymal crosstalk (e.g. Wnt and Shh signaling) to drive appendage growth, and fetal immune colonization to establish homeostasis [50]. In contrast, aging reflects the effects of heterogeneous tissue degeneration under intrinsic and extrinsic stressors [57]. Traditional research has been restricted by ethical limits related to human embryonic studies and the inability of *in vitro* models to replicate the complex *in vivo* niche [58]. With its capacity for *in situ*, multi-time point, single-cell resolution, spatiotemporal omics can be used for the reconstruction of multidimensional atlases of skin and the dynamic mechanisms involved from embryonic development to physiological aging (Figure 4a and b) [59–70].

#### Skin development

##### Epidermal development

During development, the epidermis is generated from the surface ectoderm as a single layer of unspecified progenitors that later give rise to the interfollicular epidermis (IFE), HFs, sebaceous glands, and mechanosensory Merkel cells [71, 72]. The IFE constitutes a continuous multilayered keratinized epithelium between HFs and serves as the principal skin barrier. It comprises a basal layer of K14^+^/K5^+^ basal cells and multiple suprabasal layers of terminally differentiated K1^+^/K10^+^ keratinocytes that progressively lose their nuclei and are shed as corneocytes [73]. Throughout the organismal lifespan, the IFE maintains epidermal renewal and homeostasis via the continuous proliferation of stem and progenitor cells. scRNA-seq analysis of early mouse embryos [embryonic day (E) 12.5–14.5] integrated with pseudotime and RNA velocity analyses revealed that although the IFE is still morphologically unilaminar at E12.5, molecular heterogeneity has already arisen, with the cells segregated into a Tagln^+^ signaling-active cluster and a Krt14^+^ basal cluster. With CellChat [17], a tool used to infer CCC networks based on curated ligand–receptor interactions, the upstream signaling programs driving this process were further delineated, and the periderm was identified as a key developmental signaling hub. Rather than serving as a passive covering layer, periderm cells actively secrete Ephrin ligands and TGF-β signaling molecules to regulate basal cell proliferation and the timing of epidermal stratification [45, 59]. Regarding the progression of development into the critical stratification phase (E14.5–E16.5), RNA velocity algorithms have revealed that IFE differentiation is a continuous process involving a multitude of transitional states. Motif enrichment and recognition analyses, which are used to identify overrepresented transcription factor binding motifs along differentiation trajectories, have shown that GRHL3 is a central transcriptional regulator along this path. At birth [postnatal day (P) 0], GRHL3 directly represses Wnt signaling, inhibits basal expansion, and promotes the commitment of basal cells to differentiation and keratinization [60]. In this complex process of lineage establishment, tissue quality control mechanisms are equally crucial. Studies based on a scRNA-seq-derived cell competition scoring model have quantitatively shown that approximately 20–30% of progenitors are identified as ‘losers’ because of their lower ribosomal biosynthetic activity. These cells are subsequently engulfed and cleared by neighboring ‘winners’ before E17.5, ensuring that the skin barrier present at birth is constructed from the most metabolically robust clonal populations [61]. During the postnatal growth phase, the epidermis undergoes a period of constant mild imbalance, as revealed by Dekoninck *et al.* through multi-time point scRNA-seq and lineage tracing. Basal cells adopt a highly biased self-renewal strategy, with symmetric proliferative divisions accounting for approximately 62% of divisions, significantly exceeding the percentage of differentiative outcomes. This skewed division mode maintains the stem/progenitor pool during the rapid tissue expansion occurring during the first 1–2 months after birth [74]. Finally, by integrated scRNA-seq and ST profiling of human fetal skin (postconceptional week (PCW) 7–17), the developmental trajectory of human IFE was reconstructed, identifying POSTN^+^ basal cells that diverge toward outer root sheath (ORS) or inner root sheath (IRS) lineages [50]. These datasets provide the first spatiotemporal framework for deciphering human epidermal morphogenesis and establish a foundation for future multiomics studies of early skin development.

##### Appendage development

Skin appendages, including HFs, sebaceous glands, and sweat glands, arise via reciprocal epithelial–mesenchymal interactions between the epidermis and dermis [75]. Among these appendages, the HF is the most archetypal and structurally sophisticated and is characterized by a highly organized 3D architecture. The ORS extends from the basal epidermis, whereas the IRS arises from the hair matrix. The dermal papilla (DP) develops from dFBs. The lower bulbous compartment, encompassing the hair germ and bulge niche, harbors an abundance of hair follicle stem cells (HFSCs) that preserve the long-term regenerative capacity and stemness properties of the follicle. HF development during embryogenesis proceeds through three stages: induction, organogenesis, and cytodifferentiation. In adulthood, the HF undergoes a cyclic regeneration process involving the telogen, anagen, and catagen phases [76]. These cycles drive rapid tissue turnover and highlight the dynamic nature of HF biology. Resolving lineage characteristics across developmental stages requires spatiotemporal resolution.

Using the CUBIC protocol, volumetric visualization of full-thickness dorsal skin across the hair cycle in mice has been achieved: biopsies collected in telogen and anagen were cleared and subjected to immunolabeling/lipid staining, and HFs and sebaceous glands were subsequently resolved at single-cell resolution by volume reconstruction on confocal image stacks [77]. In the same year, Joost *et al.* [13] initiated the first quantitative characterization of cellular heterogeneity across distinct HF compartments by employing low-throughput scRNA-seq combined with a pseudo spatial inference framework. In 2020, the team improved their methodology by integrating high-throughput scRNA-seq, dynamic RNA velocity analysis, and smRNA-FISH validation of spatial localization [46]. This approach clearly identified two transcriptionally distinct cell populations—basal and suprabasal—within the ORS. Leveraging RNA velocity analysis with Markov chain modeling, they pinpointed Msx2^+^ uncommitted progenitors as the common ancestral cellular source for multiple inner lineage trajectories. Moreover, epidermal basal layer subpopulations, which critically determine HF developmental potential, were further delineated using RNA velocity analysis combined with scEpath [78], a trajectory inference framework, revealing their lineage differentiation hierarchies. HFSCs and their niche, which includes neighboring cells and the ECM, coordinate morphogenesis and regeneration through signaling interactions. Spatiotemporal omics approaches excel in reconstructing the dynamic regulatory landscape from HF formation to maturation. For example, during the placode stage of HF development in mice (E14.5), BMP signaling exhibits a sharp boundary-restricted spatial distribution, where Sox9^+^ peripheral cells locally express Bmp2 and Sostdc1, synergistically activating the Wnt pathway to drive early lineage bias. In mature HFs, pSMAD1 signaling remains highly active within the HFSC compartment, maintaining stemness while preventing premature differentiation [59, 79, 80]. Collectively, these findings reveal the continuous role of BMP/pSMAD1 signaling in early fate determination and subsequent stemness maintenance during HF development. Furthermore, Wang *et al.* [81], who employed a porcine model more physiologically relevant to humans than are mouse models, performed cross-species comparative analyses integrating human, mouse, and porcine HF developmental datasets. By employing cutting-edge tools such as Monocle 2, which reconstructs pseudotime differentiation trajectories, in combination with CytoTRACE, a framework that estimates cellular differentiation potential on the basis of transcriptional diversity, Wang *et al.* identified an OGN^+^/UCHL1^+^ population as early-placode-stage progenitors whose lineage fate is jointly governed by BMP and TGF-β signaling.

##### Mesenchymal development

Originating from dual lineages (mesoderm and neural crest cells), the dermal mesenchyme provides the essential mechanical and signaling framework for skin morphogenesis [82–84]. The central orchestrators of mesenchymal development, heterogeneous dFBs specify the skin microenvironment and are governed by hierarchical differentiation programs that pattern the vasculature, adipose tissue, and HFs. Early studies using genetic lineage tracing revealed a key fate transition at E16.5 in mice. Activation of Wnt/β-catenin signaling drives upper dermal cells toward the DP, arrector pili muscle (APM), and papillary fibroblast lineages, whereas silencing of this pathway in lower dermal cells results in the development of the reticular dermis, subcutaneous tissue, and dermal white adipose tissue (dWAT) [85, 86]. Via spatiotemporal omics, the molecular logic of this process has been further decoded at single-cell resolution. Using RNA velocity analysis and CellRank, a framework that integrates directional dynamics with cell–cell similarity to map fate potential, Jacob *et al.* identified a fibroblast-originating subpopulation (FIB Origin cells) in mouse embryonic skin at E12.5 [45]. Pseudotime analyses revealed that fascia and adipocyte lineages arise at E13.5, earlier than the conventionally accepted E16.5 timeframe. In this study, along with related work [62], CellChat was used to reconstruct intercellular signaling networks, revealing a close link between pathway activity and lineage decisions. These findings challenge the conventional view that adipocyte and fibroblast lineages are entirely independent, showing that a lower dermal subpopulation (HI-Aps cells) possesses bidirectional plasticity, with its fate transitions dynamically regulated by the balance between Wnt and IL-1 signaling. Recent studies integrating spatial metabolomics with scRNA-seq analysis have revealed that metabolic state remodeling plays an essential role in lineage specification. For instance, Capolupo *et al.* [63] discovered that spatial heterogeneity in sphingolipid metabolism is closely associated with hierarchical fibroblast differentiation in the DP and reticular layers. Building on this concept, a recent study integrating scRNA-seq, ST, and spatial metabolomics demonstrated that the glycosphingolipid Gb3, via the HEXB–Gb3–FGF2 axis, differentially regulates the papillary and reticular fibroblast differentiation trajectories following superficial and deep second-degree burns, respectively [87]. Compared with transcriptomic profiles, chromatin accessibility has stronger predictive power for lineage restriction and terminal fate decisions [88]. In recent studies employing single-cell ATAC using sequencing (scATAC-seq) combined with Signac, an R package specifically developed for the analysis of chromatin accessibility data, differential accessibility region analysis, and gene activity matrix modeling, the epigenetic regulatory patterns of fibroblast lineage specification during skin development have been dynamically mapped [89–91]. These studies revealed that the binding motifs of transcription factors such as Runx1, Twist2, and AP-1 (Jun/Fos) exhibit differential openness across lineages, suggesting that chromatin remodeling precedes transcription in defining the differentiation potential of dFBs. With the integration of posttranscriptional regulation by RNA-binding proteins [92], epigenetic modulation, and metabolic reprogramming in future research, a more precise and mechanistic understanding of terminal lineage fate determination in dFBs is expected.

##### Immune system development

The largest barrier organ, the skin depends on early immune system establishment to maintain function and adapt to environmental changes. The ontogeny of skin immunity is intricately intertwined with skin-intrinsic cell/matrix and appendage development, which provides crucial regulatory signals for the patterning and maintenance of skin architecture. Cytometry by time-of-flight (CyTOF), a high-dimensional proteomics platform, can be used to detect multiple functional proteins simultaneously [93]. It is particularly suited for identifying immune cell phenotypes, activation states, and functional heterogeneity, thereby complementing scRNA-seq by addressing its limitations at protein-level resolution. Using CyTOF integrated with the FlowSOM clustering algorithm, Dhariwala *et al.* [94] systematically revealed the presence of CD4^+^ T cells, CD8^+^ T cells, and functionally mature FOXP3^+^ regulatory T cells (Tregs) in second-trimester human skin. These Tregs preferentially accumulated near developing HFs and established an immune-tolerant microenvironment, partly via the secretion of factors such as TGF-β, to prevent autoimmune responses against nascent HF antigens, thereby ensuring successful morphogenesis. Further integration of this approach with 16S rRNA sequencing demonstrated that this immune preprogramming is regulated by intrauterine microbial signals [95]. Trace microbes (e.g. *Staphylococcus* spp.) present in the fetal skin and gut are captured by antigen-presenting cells (APCs), inducing the early priming and expansion of CD4^+^ T cells via the major histocompatibility complex–T-cell receptor (MHC–TCR) signaling axis. This observation suggests that the immune system in the fetal skin acquires primary immune memory through microbe–immune interactions before birth, which in turn prepares the organism for postnatal environmental adaptation. In contrast to this integrated approach, scRNA-seq offers unique advantages in resolving lineage trajectories and spatial hierarchies, making it more suitable for studying earlier embryonic windows. For example, Zhao *et al.* [64] traced the origin of mast cells to Cpa3^+^ yolk sac-derived progenitors emerging at PCW 9–12, challenging the canonical view of their bone marrow-derived origin. Similarly, Reynolds *et al.* [44] constructed a comprehensive multiorgan single-cell atlas spanning PCW 7–10, systematically reconstructing the colonization dynamics of macrophages, Langerhans cells, and dendritic cells (DCs). By applying the TransferAnchors algorithm in Seurat, which maps shared cellular features across datasets, they integrated data from fetal and adult skin, enabling the quantitative assessment of the immune cell composition across developmental stages. Researchers have employed Cell2location, a Bayesian model for inferring cell type abundance from ST data using single-cell references, in combination with differential abundance analysis (DESeq2 + limma). This approach enabled the construction of a high-resolution spatiotemporal atlas of human skin immune development covering PCW 7–17, providing a reference for the distribution and colonization of immune cells in skin [50].

#### Skin aging

##### Identification of aging biomarkers

Skin aging is characterized by progressive structural and functional alterations, such as wrinkle formation, reduced elasticity, hyperpigmentation, dermal thinning, and chronic low-grade inflammation [96, 97]. Cellular senescence is a central event and is marked by transcriptional transitions mediating processes from stemness exhaustion to inflammatory remodeling [65]. The transcriptional and secretory profiles associated with senescence have thus become central to understanding the mechanisms of tissue degeneration and designing antiaging interventions [98]. The integration of scRNA-seq with spatial omics has opened new avenues for the refined characterization of skin aging biomarkers, moving beyond reliance on single-gene markers toward multigene and multipathway integration [99]. Among various skin cell types, fibroblasts exhibit the greatest degree of senescence-associated transcriptional heterogeneity. In scRNA-seq analyses of human eyelid skin across different age groups, HES1 was identified as a key antiaging regulator [66]. In young fibroblasts, HES1 recruits histone deacetylase complexes to directly repress CDKN1A (p21) promoter activity, thereby maintaining cell cycle control. In contrast, the loss of HES1 in aged fibroblasts leads to deregulated CDKN1A expression, mitochondrial dysfunction, and the aberrant accumulation of reactive oxygen species. A study with Monocle 2 further revealed senescence-induced arrest in the differentiation trajectory of basal cells within the epidermis. In young skin, keratinocytes follow a continuous basal to spinous and spinous to granular trajectory. In aged skin, this trajectory is disrupted and stalled, indicating a reduced differentiation potential rather than a complete loss of hierarchy. Further analyses demonstrated significant downregulation of stemness markers (Sox9 and Lhx2) in aged basal cells, accompanied by upregulation of inflammatory pathway-related genes (Stat3 and Junb) [65]. Recently, the development of novel algorithms and gene sets such as IRescue [100], SenPred [101], and SenSkin [102] has substantially improved the ability to detect early senescence states and capture functional transitions, overcoming the limitations of relying on CDKN2A (p16) as a single-gene marker. These scoring systems enable quantitative staging of skin aging and offer actionable metrics for the development of targeted therapies.

##### Senescence-driven cell–cell communication and spatial remodeling

Although senescent cells lose their proliferative capacity, they retain their active secretory functions. The set of senescence-associated secretory phenotype (SASP) factors these cells produce is rich in proinflammatory cytokines, chemokines, and matrix-degrading enzymes. Through paracrine signaling, SASP factors spread senescence to neighboring cells, amplifying degeneration [103, 104]. Through the use of CCC analysis tools such as CellChat and NicheNet, spatiotemporal omics studies have revealed the pivotal role of senescent cells in the remodeling of the skin microenvironment.

On the one hand, skin aging is closely linked to prolonged HF cycling. Studies have revealed that the attenuation of signaling interactions between DP cells and ECs in aged skin is a key mechanism underlying impaired hair regeneration. This loss of signaling was substantiated by scRNA-seq combined with CellChat analysis. By integration of this approach with the scMetabolism tool and iPATH, a metabolic pathway visualization tool, downstream effectors such as taurine (TA) and α-linolenic acid (ALA) were identified, and these effectors were validated *in vivo* as potential therapeutic targets [105]. On the other hand, immune cells further drive senescence by inducing chronic inflammation. scRNA-seq analyses have revealed that aged skin contains elevated populations of dermal CD4^+^ T cells, γδ T cells, and innate lymphoid cells (ILCs), which secrete large amounts of IL-17 that activate NF-κB signaling in keratinocytes and disrupts skin homeostasis. Anti-IL-17 therapy was found to attenuate inflammatory damage and restore the expression of approximately 5317 homeostatic epidermal genes, as confirmed by comparisons of scRNA-seq data acquired before and after treatment, demonstrating the reversibility of immune–epidermal communication circuits [106]. Similarly, the natural compound Lapagyl was found to exhibit antiaging effects by blocking CCL8-mediated immune crosstalk, as validated through multiomics profiling across the treatment duration [107]. Furthermore, ST addresses the limitations of scRNA-seq caused by the loss of fragile senescent fibroblasts during tissue dissociation, preserving critical spatial information [108]. By ST in combination with the Cell2location algorithm, the spatial clustering patterns of inflammatory cells and stem cells within aged skin were reconstructed, revealing that regional structural degeneration driven by the immune microenvironment constitutes a key hallmark of skin aging [109]. Data from the 10x Genomics Visium platform demonstrated that CDKN1A-high senescent cells form pronounced spatial clusters in photoexposed skin, with the formation of these clusters significantly exceeding that in both nonphotoexposed regions and randomized spatial simulations. These findings provide additional corroboration of the paracrine diffusion effect of the UV-induced SASP [6]. These spatially localized senescent hotspots may constitute the histological basis underlying region-specific aging phenotypes such as pigmentation patches and solar lentigines.

### Revealing pathology: spatiotemporal omics breakthroughs in skin diseases

Spatiotemporal omics has revolutionized the understanding of skin diseases through the dissection of pathological processes at single-cell, spatial, and temporal resolutions. In chronic inflammatory disorders such as atopic dermatitis (AD) and psoriasis, these technologies have revealed developmental gene reactivation, complex CCC networks, molecular subtypes, and metabolic vulnerabilities, offering refined diagnostic frameworks and identifying novel therapeutic targets. In the context of autoimmune conditions, spatiotemporal profiling has revealed widespread immune dysregulation extending beyond lesional sites, highlighting the systemic involvement in these conditions and the predictive value of nonlesional skin. Applications to malignant skin diseases—including melanoma, basal cell carcinoma (BCC), and cSCC—have included the mapping of tumorigenic trajectories, the evaluation of tumor–stroma–immune interactions, and the identification of mechanisms of therapeutic resistance within the tumor microenvironment (TME). Collectively, these advances highlight spatiotemporal omics as a transformative paradigm for decoding cutaneous pathogeneses, refining molecular classifications, and guiding precision therapies in benign, autoimmune, and malignant skin disorders (Figure 4c).

#### Benign skin diseases

##### Chronic inflammatory skin diseases

AD and psoriasis are the most representative chronic inflammatory skin diseases. AD features a Th2/Th22-dominated immune bias that is typically associated with epidermal barrier dysfunction and chronic inflammation [110]. Psoriasis is driven primarily by Th1/Th17 immune responses and is characterized by excessive basal cell proliferation and abnormal KC differentiation, with manifestations including parakeratosis and hyperkeratosis [111]. As a 3D structural reference, BABB-based OTC combined with light sheet fluorescence microscopy (LSFM) has enabled comprehensive visualization of psoriatic lesions, revealing marked epidermal thickening with focal parakeratosis and spongiotic changes with loss of the granular layer, accompanied by widespread inflammatory infiltration in the dermis [112].

Spatiotemporal omics approaches have been increasingly applied in studies of AD and psoriasis, extending beyond disease-associated cell type annotation to encompass mechanistic dissection, molecular subtyping, metabolic target discovery, and therapeutic response evaluation. In mechanistic studies, integrated multiomics and pseudotime analysis revealed that keratinocytes, ECs, and macrophages drive pathological processes in both AD and psoriasis by reactivating embryonic developmental programs [44]. Furthermore, joint analyses using scRNA-seq and ST revealed intricate CCC networks in inflammatory skin diseases. For instance, tissue-resident ILCs were found to drive lesion formation in psoriasis, whereas COL18A1^+^ fibroblasts in AD lesions were found to engage in intense crosstalk with immune cell populations [113, 114]. With respect to diagnostics, researchers have established RashX, a scRNA-seq-based CD45^+^ immune cell atlas platform, to aid in the classification of clinical/histopathologically indeterminate rashes (CIRs) as AD or psoriasis vulgaris (PV), demonstrating the potential of single-cell molecular pathology in clinical decision-making [67]. ST has enabled a more granular, spatially informed molecular stratification of psoriatic lesions that complements conventional Psoriasis Area and Severity Index (PASI)-based phenotypic scoring. By mapping the spatial colocalization of TNF^+^ CD8^+^ T cells, IFI6^+^ keratinocytes, and CXCL13^+^ fibroblasts, psoriatic lesions were classified into molecularly distinct subgroups, providing a more refined diagnostic framework [115]. In terms of clinical intervention and target discovery, the results of integrated scMetabolism analyses have highlighted metabolic reprogramming as a core driver of psoriasis pathogenesis. The psoriatic epidermis exhibits a unique metabolic stratification: basal cells activate a high-intensity glycolytic program via HIF1α-mediated transcriptional regulation, which not only fuels excessive proliferation but also causes glucose deprivation in suprabasal layers, subsequently inducing disulfide stress and cytoskeletal remodeling [116]. More importantly, recent research revealed a pathological epithelial-immune metabolic circuit. IL-17 induced the upregulation of HIF1α expression and glycolytic activity in epidermal cells, leading to the secretion of the metabolic byproduct lactate. This lactate was then captured by dermal γδ T17 cells via the MCT1/4 transporters, which in turn promoted T-cell survival and sustained IL-17 production [68]. These findings establish HIF1α and lactate transporters as critical drug targets for disrupting the inflammatory cycle in psoriasis. The results of *in vitro* experiments confirmed that HIF1α inhibitors exhibit anti-inflammatory efficacy comparable to that of IL-17 inhibitors, the current first-line clinical treatment [117]. Next-generation spatiotemporal omics platforms have emerged to address the clinical challenges of heterogeneity and subtype classification in inflammatory dermatoses. For example, Seq-Well S^3^ increases transcript capture and gene detection efficiency by approximately 10-fold and 5-fold, respectively. This improvement enables the preservation of low-abundance transcription factors, cytokines, and their receptors, which were previously difficult to detect, in lymphocytes. This technology has been applied to generate high-resolution transcriptional atlases of 5 human inflammatory skin disorders—acne, alopecia areata (AA), granuloma annulare, leprosy, and psoriasis. These datasets provide important resources for systematically dissecting the mechanisms of cutaneous immune disorders [118]. Several studies have specifically investigated the mechanisms of pathological pruritus in AD and psoriasis, which are linked to microstructural alterations in intraepidermal nerve fibers (IENFs) [119, 120]. By combining OTC with the suction blister technique to isolate an intact epidermal sheet, IENFs can be recovered en bloc and reconstructed, enabling standardized morphometric assessment and offering a complementary route for spatial omics investigations [121].

##### Hair-related disorders

Hair-related disorders encompass a broad spectrum of phenotypes, ranging from nonscarring alopecia [e.g. AA and androgenetic alopecia (AGA)] and scarring alopecia [e.g. acne keloidalis (AK)] to age-related hair loss and canities (hair graying) [122–124]. Despite their apparent clinical heterogeneity, these conditions share common mechanistic features at the molecular level—namely, disruption of the immune–epithelial–stromal signaling network within the HF stem/progenitor cell niche.

Through scRNA-seq and ST, the specific mechanisms of perifollicular inflammation *in situ* have been elucidated. Skin-resident Tregs are a key immune population that maintains immune privilege within the HFSC niche. They suppress perifollicular effector T cells through the competitive consumption of IL-2, thereby protecting HFs—particularly HFSCs—from autoimmune destruction. Disruption of this Treg–IL-2 axis within the perifollicular stroma triggers the pathological immune attacks characteristic of AA and scarring alopecia [125]. In the late stages of AK, granulomatous reactions and extensive fibrosis occur. A CellChat analysis linked POSTN^+^/ASPN^+^ fibroblasts with SPP1^+^/MMP9^+^ myeloid cells via a verifiable SPP1–CD44 signaling axis. Furthermore, by Visium-based ST utilizing module-score projection and pretreatment/posttreatment comparisons, these cellular states were demonstrated to have distinct microanatomical localizations (proximal to broken HFs and fibrotic zones) and to be correlated with lesion activity and glucocorticoid efficacy [126]. Studies on AA have further proven that this immune dysregulation is not localized to the follicle. Analysis of integrated scATAC-seq and transcriptomic profiling data revealed that peripheral blood mononuclear cell (PBMC) subpopulations in AA patients exhibit proinflammatory transcriptional signatures and altered gene regulatory networks mirroring those of *in situ* immune cells, suggesting a role for systemic immune dysregulation in disease progression [127]. In contrast, AGA is characterized by prominent chronic microinflammation in the upper HF segments, a feature requiring higher spatial resolution for investigation. By combining ST with GeoMx digital spatial profiling (DSP), a region-of-interest (ROI)-based spatial multiomic platform, researchers precisely mapped the immune microenvironment within a 100–200 μm radius of the HF infundibulum, identifying significant enrichment of Th2 responses that directly drive fibrotic transformation in the progenitor zone [128, 129]. Building upon this finding, Ober-Reynolds *et al.* [130] generated paired scRNA-seq and scATAC-seq atlases of the human scalp, enabling cell type-resolved mapping of regulatory elements and gene programs. By integrating these maps with GWAS fine-mapping, they demonstrated that AGA-associated genetic signals are significantly enriched in regulatory elements active in HF progenitors and infundibular keratinocytes. Notably, balding scalps exhibited early epigenetic reprogramming in these progenitors, which was characterized by increased accessibility of binding sites for inflammatory transcription factors (e.g. members of the AP-1 and STAT families) and reduced accessibility of WNT-associated regenerative elements, suggesting a shift toward a proinflammatory, regeneration-impaired chromatin state preceding overt follicular miniaturization. The natural regression of hair cycling—exemplified by age-related alopecia and graying—primarily involves shifts in cell fate. In aged mouse models, RNA velocity analysis revealed that hair loss is not due solely to HFSC exhaustion; rather, hair follicle dermal stromal cells (hfDSCs) develop functional impairments driven by dysregulated AP-1 activity and Cyr61 (CCN1) overexpression [131]. These events lead to a diminished capacity for differentiation into DPs, ultimately resulting in follicular atrophy. With respect to hair graying, analyses with Monocle 3 and Slingshot, two trajectory inference tools for pseudotime analysis that extend and complement earlier Monocle 2-based lineage modeling, revealed that in addition to the depletion of melanocyte stem cells (McSCs), the specific loss of matrix hair progenitors is a critical early event and is accompanied by the aberrant activation of the P53 signaling pathway [132].

##### Other benign diseases

In addition to AD and psoriasis, vitiligo has emerged as a key model for spatiotemporal omics research aimed at elucidating the mechanisms of autoimmune microenvironmental remodeling. Vitiligo is a prototypical autoimmune skin disorder in which T-cell–mediated melanocyte-specific cytotoxicity drives disease progression. In particular, CD8^+^ T-cell–mediated local attacks lead to targeted melanocyte depletion and the subsequent formation of depigmented patches [133, 134]. Recent scRNA-seq studies of vitiligo lesions and adjacent clinically normal skin have revealed a critical immune tolerance checkpoint dependent on the CCR5–CCL5 axis [135]. In nonlesional skin, Tregs are not quiescent but instead actively sense inflammatory cues through type 1 cytokine signaling and upregulate CCR5 expression, which enables their chemotactic localization near CCL5-producing effector CD8^+^ T cells and thereby allows them to exert localized immunosuppressive control. During the progressive stage of vitiligo, the loss of CCR5 expression on Tregs causes this spatial colocalization mechanism to fail, allowing effector T cells to escape immune surveillance and destroy melanocytes. Extending these analyses to PBMCs using integrated scRNA-seq and scATAC-seq data, researchers have identified specific chromatin remodeling features in the peripheral immune cells of vitiligo patients, suggesting that the systemic immune imbalance is preprogrammed by epigenetic modifications [136]. These systemic chromatin-level alterations imply that the risk of vitiligo pathogenesis may already be imprinted in the genomic regulatory elements of circulating immune cells, underscoring the necessity of systemic immunomodulatory therapies. Notably, molecular profiling of nonlesional skin has provided direct guidance for early intervention and drug development. The upregulation of Th2 pathway-associated genes, including IL4R, CCL17, and IL13, in nonlesional areas suggests that these pathways may play dual roles in early lesion formation and therapeutic targeting [137]. These findings offer a mechanistic explanation for why exclusive blockade of Th1 signaling may be insufficient to fully control disease activity, whereas broader-spectrum agents such as JAK inhibitors—which simultaneously target Th1 and Th2 pathways—have demonstrated superior clinical efficacy. Therapeutic modulation of Th2-associated programs in nonlesional skin may therefore constitute a promising strategy to prevent the transition from subclinical immune dysregulation to the development of overt depigmented lesions. In contrast, spatiotemporal omics research in other nonneoplastic skin conditions—such as melasma, lichen planus, seborrheic dermatitis, rosacea, acne, and cutaneous lupus erythematosus—remains in a preliminary exploratory phase. This limited progress stems from several challenges, including limited lesion accessibility, the difficulty of obtaining high-quality tissue samples, and the lack of well-defined cell type annotations or pathogenic frameworks. Consequently, most studies in these diseases remain confined to bulk RNA-seq, which cannot resolve cellular heterogeneity, spatial microenvironmental remodeling, and dynamic disease trajectories.

#### Malignant skin diseases

##### Spatial behavior of tumors

Tumors often originate from precursor clones residing within specific anatomical niches, and their progression is tightly regulated by spatiotemporal dynamics [138, 139]. In melanoma, large-cohort analyses comparing *in situ* and invasive acral subtypes using scRNA-seq combined with ST revealed the enrichment of APOE^+^/CD163^+^ macrophages surrounding tumor cells undergoing epithelial–mesenchymal transition (EMT) mediated by the IGF1–IGF1R signaling axis, suggesting that an immunosuppressive microenvironment plays a critical role in driving invasion. These findings were independently validated at the protein level using CODEX-based spatial proteomics [140]. The results generated via this integrative multiomics approach underscore the importance of identifying subclonal mutations and spatially mapping immune cell distributions within early-stage melanoma lesions. In BCC, spatiotemporal omics analyses have further revealed how the physical microenvironment regulates tumor fate through mechanotransduction signaling. Integrated scRNA-seq and AUCell-based regulon activity analysis demonstrated that in soft matrix regions (e.g. the auricle), the low stiffness inhibits YAP/TAZ activity, thereby increasing the repression of the embryonic hair follicle progenitor (EHFP) transcriptional program and endowing tumor cells with potent vertical invasive capacity. Conversely, in stiff matrix regions (e.g. the back), high YAP/TAZ activity inhibits this reprogramming, limiting growth to lateral expansion [141]. Although ST was not employed in that study, distinct competitive and migratory behaviors among tumor cell populations were successfully identified by integration of intravital spatial imaging. In cSCC, single-cell clustering was further refined by ST to precisely localize tumor-specific keratinocyte (TSK) subpopulations within fibrovascular niches at the invasive tumor front. These TSK clusters exhibited significant spatial colocalization with Tregs and CD8^+^ T cells, indicating that they function as structural hubs that facilitate tumor invasion [16]. Similarly, spatiotemporal omics has been applied to delineate the unique spatial architecture of cutaneous T-cell lymphomas (CTCLs), with the results revealing that B-cell infiltration strongly correlates with disease progression and patient prognosis, suggesting the potential utility of B-cell infiltration as a prognostic biomarker [142]. Interestingly, tumors of noncutaneous origin but with prominent cutaneous manifestations also demonstrate well-defined spatiotemporal evolutionary trajectories. For example, in blastic plasmacytoid dendritic cell neoplasm (BPDCN), integrated analyses combining lineage tracing, scRNA-seq, and XV-seq were used to map the sequential progression from premalignant clonal expansion in the bone marrow to UV-induced mutation acquisition in the skin followed by reverse metastatic seeding to the bone marrow [143]. This dynamic reconstruction highlights the unparalleled value of spatiotemporal omics in reconstructing tumor development dynamics, rendering each step of tumor evolution ‘visible.’

##### Targeted immunotherapeutic strategies

The TME, which is composed of diverse cell populations and the ECM, plays a central role in driving tumor initiation, progression, immune evasion, and therapeutic resistance [144]. A complete understanding of the 3D architecture of the TME is essential to ensure the full removal of cutaneous tumors during excision procedures such as Mohs surgery [145]. In the latest Skin-iDISCO^+^ protocol for human skin, acid-mediated collagen denaturation is replaced with efficient depigmentation, which improves optical transparency and imaging signals while preserving vascular/adnexal scaffolds and reducing network distortion [146]. These advances have provided a high-fidelity 3D spatial reference for the precise delineation of surgical margins. Emerging evidence indicates that the immune microenvironment is a key determinant of antitumor immunity that potentially explains the frequent clinical failure of immune checkpoint blockade (ICB) therapy in certain patients [147]. Spatiotemporal omics approaches support the exploration of resistance mechanisms and the development of corresponding targeted strategies through comparison of the differing immune response states in pre- and posttreatment samples. For instance, scRNA-seq revealed 9 distinct posttreatment tumor cell states in ICB-treated melanoma patients. CellTrek is a computational algorithm that precisely maps scRNA-seq data onto ST coordinates, effectively overcoming the limited resolution of spatial platforms by projecting individual cells back onto their native tissue locations [148]. By integrating ST with CellTrek analysis, researchers revealed that mesenchymal-like (MES) tumor cells possess a spatial advantage for immune evasion. SCENIC analysis revealed that TCF4 is the master regulator that promotes the MES program while suppressing melanocyte differentiation and antigen presentation [149]. Crucially, TCF4 expression depends on a BRD4-bound enhancer region—utilizing the BET degrader ARV-771 to reduce TCF4 levels resulted in the downregulation of MES genes and upregulation of antigen presentation/IFN response genes while increasing sensitivity to ICB and targeted therapies. These findings identify a therapeutically actionable TCF4–BRD4–epigenetic enhancer axis for reversing immune tolerance. Similarly, in Merkel cell carcinoma (MCC), which has an ICB response rate of approximately 50%, multimodal integration of scRNA-seq, multiplex immunofluorescence, and ST data revealed that spatially organized immune triplets—comprising CD8^+^ tissue-resident memory T (Trm) cells, B cells, and DCs—serve as the structural basis for effective immune responses. Survival analysis demonstrated that a higher Trm/Tcirc (circulating T) cell ratio was correlated with a more favorable treatment response and a longer patient survival time [150].

### Illuminating repair and regeneration: spatiotemporal insights into wound reconstruction

Upon skin injury, fibroblasts transition from a quiescent state to an activated state, exhibiting lineage-specific migratory, proliferative, and differentiation capacities and performing distinct functions during different stages of repair. Wound healing is a highly dynamic and tightly spatiotemporally regulated process. Spatiotemporal omics provides a new paradigm for evaluating wound healing, scar formation, skin appendage regeneration, and tissue engineering at unprecedented resolution (Figure 4d).

#### Wound healing

Wound healing is divided into three overlapping stages—inflammation, proliferation, and remodeling—and requires coordinated transitions among diverse cell populations. Spatiotemporal omics techniques, particularly scRNA-seq, have revealed that during the inflammatory phase, the monocyte/macrophage (MΦ) lineage initially exhibits a proinflammatory phenotype to mediate antimicrobial responses, subsequently transitioning toward a reparative state [151]. This phenotypic switch is accompanied by metabolic reprogramming characterized by a shift from glycolysis to oxidative phosphorylation. Moreover, scRNA-seq has revealed the reparative potential of rare immune populations, such as mucosal-associated invariant T (MAIT) cells. Upon migration into the wound bed, MAIT cells secrete amphiregulin to activate EGFR signaling, which in turn facilitates KC migration and re-epithelialization [152].

Upon entry into the proliferative phase, fibroblasts become the principal drivers of tissue repair. By integrating scRNA-seq, scATAC-seq, and ST data, researchers established a spatial epigenomic inference framework to resolve the tissue-scale dynamics of chromatin accessibility [153]. Using ArchR in combination with GREAT and CytoTRACE, this multimodal analysis identified a mechanosensitive fibroblast subpopulation that migrated directionally from the wound edge toward the center. This subpopulation was characterized by increased accessibility of YAP/TAZ- and TEAD-associated regulatory elements, accompanied by the activation of genes involved in ECM production and cytoskeletal remodeling, thereby linking mechanical cues to fibroblast fate decisions. Further scATAC-seq analysis revealed that distinct fibroblast lineages are epigenetically preprogrammed, which in turn defines their functional potential during repair. For example, Hic1^+^ fibroblast lineages exhibit chromatin accessibility and transcription factor-binding profiles closely resembling those of the embryonic inductive mesenchyme, accompanied by increased responsiveness to the Wnt, FGF, and BMP signaling pathways [154]. These findings suggest that a subset of fibroblasts retain latent competence to support appendage-related structural programs within the wound environment, with functional outcomes constrained primarily by local signaling cues rather than intrinsic incapacity. In models of severe injury, such as burn wounds, time-resolved scATAC-seq analysis further revealed dynamic regulatory mechanisms governing fibroblast responses during acute injury, including the stage-specific activation of AP-1–, NF-κB–, and TGF-β–associated transcriptional networks [155]. Collectively, the findings of these studies underscore the idea that fibroblasts do not maintain static subtypes but continuously remodel their functional states under the combined influence of mechanical forces, inflammatory signals, and epigenetic regulation. Chronic wounds, which are clinically classified into three main categories—leg ulcers (venous/arterial deficiencies), diabetic foot ulcers (DFUs), and pressure ulcers—are typically characterized by persistent inflammation and impaired angiogenesis [156]. By integrating scRNA-seq with ST, researchers mapped the cellular landscape of DFU wounds and revealed that the spatial co-enrichment of HE-Fibro subpopulations and M1 macrophages is critical for wound healing [157]. In another study, angiogenesis-associated differentially expressed genes in human dermal microvascular endothelial cells (HDMECs) were identified, and in a subsequent screen, RAB17 was identified as a potential therapeutic target via LASSO regression [158]. More recently, integrative multi-time point analyses combining scRNA-seq data with ST data from acute and chronic wounds, supported by trajectory inference and Cell2location analysis, revealed chronic wound-specific dysregulated regulatory nodes. These included failure to appropriately resolve inflammatory signaling, arrest of fibroblasts at intermediate ECM remodeling states, and insufficient activation of angiogenesis-related transcriptional programs [159]. Collectively, these findings underscore the idea that chronic wounds do not develop because of dysfunction of a single cell type but instead result from system-level spatiotemporal imbalance across multicellular regulatory networks. In parallel, *in vivo* OTC-enabled two-photon and spectral imaging has allowed continuous, label-free assessment of collagen remodeling and microvascular permeability [160]. This 3D structural framework supports the staging and response evaluation of chronic wounds and can be used to cross-validate molecular readouts from spatial omics. Certain studies have further elucidated systemic regulatory mechanisms. Clearance of senescent cells at wound edges was found to accelerate wound closure by restoring the reparative capacity of neighboring fibroblasts [161], whereas thyroid hormone signaling was found to precisely orchestrate the temporal coordination of epidermal repair and dermal remodeling [162]. These investigations signify a new phase in wound healing research that is being propelled by spatiotemporal omics from local microenvironmental studies to systemic analyses of endocrine regulation. This shift is particularly relevant in the context of severe injuries such as burns, whose pathophysiology often involves a hypermetabolic state affecting multiple organ systems [163], underscoring the importance of multilayered analyses spanning the local and systemic levels.

#### Scar formation

Scarring is the predominant outcome of wound healing in adult mammals and is characterized by dense collagen deposition, loss of vasculature and skin appendages, and impaired tissue function compared to that in normal skin. Myofibroblasts function as the principal effector cells during the remodeling phase, where their persistent activation and collagen synthesis directly drive fibrotic scarring [164, 165]. Understanding the origins, lineage trajectories, and fate-determining mechanisms of myofibroblasts has long been a central focus in skin wound repair research [166]. Spatiotemporal omics has facilitated the construction of high-resolution fibroblast atlases, allowing the precise mapping of progenitor cell niches and spatially localized regions of hyperactivation. Early studies based on lineage tracing and flow cytometric sorting identified En1^+^ fibroblast lineages (EPFs) as the primary contributors to scar formation. These intrinsically profibrotic cells can be isolated and enriched using the surface marker CD26/DPP4, and notably, pharmacological inhibition of the enzymatic activity of DPP4 has been shown to attenuate scarring, suggesting its transformative potential [167]. Subsequently, via the integration of scRNA-seq analysis with RNAscope-based *in situ* single-cell validation and trajectory inference, a critical ‘watershed’ in activated fibroblast fate decisions within wound beds was revealed [168]: one subpopulation differentiates into a transient, reversible, repair-associated myofibroblast state, whereas the other enters a persistently activated, profibrotic program characterized by sustained collagen production. These divergent trajectories are predetermined largely by intrinsic transcriptional and epigenetic programs rather than being shaped solely by local immune or tissue environments. Although factors such as mechanical stress and inflammation can accelerate differentiation, the initial ‘preset program’ plays a decisive role. This concept was independently validated by scATAC-seq in reindeer skin regeneration models [169]. Fibroblasts entering these divergent trajectories exhibited significant differences in transcriptional states, regulatory networks, and chromatin accessibility before injury. Using high-throughput scRNA-seq on the Drop-seq platform, researchers further identified CD201^+^ fascia-derived fibroblast progenitors, a specialized EPF subset, as the primary cellular source of scar formation. PAGA trajectory analyses revealed a sequential transition from proinflammatory fibroblasts to protomyofibroblasts and ultimately to fully activated myofibroblasts [170]. Via ST, researchers localized these cells migrating from the deep wound bed toward the surface and aggregating at specific fibrotic hotspots. The diffusion pseudotime (DPT) allows the inference of continuous cellular trajectories, whereas scArches enables the cross-dataset and cross-species transfer of single-cell annotations. Integration of these approaches revealed convergent activation of retinoic acid and hypoxia-driven signaling pathways in multiple fibrotic skin diseases [e.g. systemic sclerosis (SSc) and graft-versus-host disease], suggesting potential broad-spectrum intervention targets. In addition, circulating fibroblasts, a key migratory population, can home from peripheral blood to skin tissue after injury and contribute to ECM deposition and remodeling, highlighting their potential roles in scar formation and fibrosis [171, 172]. If spatiotemporal omics could be applied to trace their dynamics from circulation to wound deposition, new evidence for their contribution to fibrogenesis may be obtained.

Excess deposition of collagen produced by these cell populations in scars results in the formation of a dense matrix that increases light scattering; accordingly, OTC is particularly needed to reduce refractive index (RI) mismatches and preserve endogenous fluorescence, enabling the application of spatial omics to obtain *in situ* 3D information from intact skin [23]. For example, a pH-adjusted BABB protocol has been validated in full-thickness wound healing and fibrosis models in mice [173]. In human keloids, head-to-head comparisons have shown that iDISCO^+^ yields greater transparency and clearer CD31 immunofluorescence than CUBIC does, allowing the confocal/LSFM-based 3D quantification of aberrant vascularization in the papillary and upper reticular dermis [174].

In addition to the effects of lineage-intrinsic programs and the deposition of secreted ECM, the development of cutaneous fibrotic disorders and diseases such as keloid and scleroderma is closely linked to their responses to the external microenvironment. Spatiotemporal omics analyses have revealed that fibroblasts are highly sensitive to mechanical and immune stimuli, which enables their reprogramming to alter their fate pathways and thereby offers potential avenues for scar intervention. scRNA-seq revealed 4 fibroblast subpopulations in keloids, with the mesenchymal-like cluster highly expressing POSTN. A study using CellPhoneDB, a ligand–receptor interaction inference framework conceptually similar to CellChat, revealed that the interaction of POSTN with integrins ITGAV/ITGB5 promotes collagen synthesis in neighboring cells [9]. In another study comparing highly invasive keloids with minimally invasive hypertrophic scars, CellPhoneDB analysis revealed that mechanosensitive fibroblasts form stronger communication links with other cells by upregulating ECM–receptor and adhesion-related pathways, in turn acting as key drivers of keloid invasiveness [175]. The latest Stereo-seq technology, which offers subcellular spatial resolution, has led to new breakthroughs in mechanistic research on fibrosis. In an SSc model, integration of Monocle 2 and MISTy spatial proximity analyses revealed that SCARA5^+^ fibroblasts exhibit spatial colocalization with CXCR4^+^ inflammatory macrophages, synergistically promoting fibrogenesis [176]. These findings not only define the spatial architecture of pathogenic niches but also pinpoint precise targets for spatially guided therapeutic interventions.

#### Wound-induced hair follicle neogenesis

In contrast to scarring repair, in which tissue continuity is restored at the expense of functional loss, wound-induced hair follicle neogenesis (WIHN) is one of the very few well-documented examples of complete regeneration in adult mammalian skin. When deep wounds exceed a critical size, the wound center is capable of regenerating fully structured HF units complete with associated sebaceous glands. These newly formed HFs are able to establish functional stem cell pools and undergo normal cycles of anagen, catagen, and telogen [177, 178].

The mechanistic investigation of WIHN began with the seminal work of Ito and colleagues [179], who first demonstrated using lineage tracing that newly regenerated HFs do not arise from residual HFs at the wound margin. Instead, they originate de novo from re-epithelialized epidermal cells in the wound center through Wnt-driven fate reprogramming. Subsequent studies employing Axin2–CreERT2 lineage tracing further revealed that Wnt-responsive Axin2^+^ interfollicular epidermal stem cells are a key cellular source for HF neogenesis and exhibit high morphogenetic potential upon receiving inductive signals from the dermis [180]. Wound size is a critical determinant of WIHN. In adult mice, small full-thickness wounds (typically circular wounds ranging between 3 and 8 mm in diameter) generally heal by forming hairless and adipose-deficient scars. In contrast, larger wounds heal via robust WIHN occurring specifically within the wound center [181]. scRNA-seq has revealed that wound size-dependent regenerative outcomes are accompanied by distinct patterns of cellular heterogeneity. WIHN-associated wounds contain an increased proportion of upper wound fibroblasts, which share transcriptional features with regenerative DP cells and migrate from the wound edge toward the center to participate in HF regeneration [182]. The *in situ* immune microenvironment that drives WIHN in large wounds has been further elucidated by ST. γδ T cells secrete FGF9, which acts on adjacent papillary fibroblasts to induce DP formation—an initiating step in HF neogenesis. In parallel, proinflammatory macrophages indirectly activate or potentiate Wnt/β-catenin signaling in neighboring epidermal cells and fibroblasts [183]. Through such coordinated spatiotemporal communication networks, de novo HFs are ultimately established. Despite these advances, studies still indicate that WIHN exhibits pronounced species-specific and translational differences. Although WIHN has been extensively characterized in mice, rats display an intrinsic deficiency in WIHN that is attributable to a reduced capacity for epidermal fate reprogramming and substantial divergence in transcriptional lineage programs [184]. In humans, WIHN is exceedingly rare and has been reported only sporadically in contexts such as deep burn wound healing or healing following BCC excision [185, 186]. However, these observations suggest that human skin cells retain latent appendage regenerative programs under specific physicochemical microenvironmental conditions. Further examination of WIHN using spatiotemporal omics will help clarify interspecies differences in regenerative capacity and identify key targets for clinically translatable strategies to promote human skin appendage reconstruction.

#### Regenerative skin engineering

The ultimate goal of skin tissue engineering is to transform bioengineered constructs into reproducible, predictable, and standardized therapeutic strategies [187, 188]. The application of spatiotemporal omics in this field offers three key advantages that enable the formation of a design–feedback–optimization positive feedback loop; these advantages are as follows. (i) Design assistance: researchers can leverage information on biomaterial heterogeneity and integrate multispecies data from public databases covering diverse wound types. Focusing on differential molecules and interacting signaling pathways allows optimized material design. For instance, Wharton’s jelly mesenchymal stem cells (WJ-MSCs) have attracted substantial attention in the field of wound repair. By scRNA-seq, WJ-MSCs were classified into 4 transcriptionally distinct subpopulations, among which the S100A9^+^CD29^+^CD142^+^ biofunctional MSC cluster exhibited superior wound healing potential. By regulatory network inference, ELK3 and RREB1 were further revealed as key upstream drivers underlying the reparative phenotype of this subpopulation. At the mechanistic level, ligand–receptor communication inference results in the identification of more specific translational targets for material/cell codesign, with signaling axes such as the AXL–GAS6 (associated with endothelial barrier repair and epithelial survival) and FZD6–WNT5A (associated with angiogenesis) axes being more prominent in this MSC subpopulation. The enrichment of this subpopulation in the fetal segment of the umbilical cord was further revealed by ST [189]. Moreover, spatiotemporal omics approaches offer *in vivo* reference blueprints for optimizing organoid-based skin models, guiding the study of hierarchical assembly logic and cellular composition ratios in the epidermis, dermis, and appendages [190]. Human amnion (hAM), exhibiting favorable mechanical properties and anti-inflammatory, antioxidant, and antimicrobial activities as well as containing growth factors that promote wound healing, has been increasingly applied in tissue engineering [191]. Bulk RNA-seq analyses have demonstrated that WJ-MSCs cultured with amnion exhibit increased immunoregulatory and reparative potential [192]. Integration of bulk RNA-seq with spatiotemporal omics could allow further elucidation of the microenvironmental mechanisms relevant to *in situ* hAM application, thereby providing more precise guidance for its clinical design. (ii) Efficacy evaluation and sensitization: spatiotemporal omics enables *in situ* characterization of microenvironmental responses to different dressings, scaffolds, microneedles, and engineered vesicles at the tissue section level. Most studies have conventionally employed bulk RNA-seq to compare molecular mechanisms in wound tissue—such as immune regulation and proliferative repair—before and after the local application of biomaterials such as hydrogels [183, 193]. In addition, in a mouse full-thickness wound model, treatment with human umbilical cord mesenchymal stromal cell-derived extracellular vesicles (hucMSC-EVs) for 7 days was found to induce a fibroblast state transition. scRNA-seq pseudotime analysis revealed that fibroblasts differentiated into an MMP13^+^ subpopulation. Analysis with NicheNetR, a ligand-receptor-based framework for predicting intercellular communication and downstream target genes, showed that these MMP13^+^ fibroblasts promote KC migration through the MMP13–LRP1 signaling axis, thereby accelerating epithelialization [69]. In another study, microporous microneedle-mediated local delivery of macrophages into full-thickness wounds in mice was found to result in a marked reduction in the number of DPP4^+^ profibrotic fibroblasts on Day 14, as revealed by scRNA-seq, indicating a promising antiscarring effect [70]. The cross-species targeting efficacy predicted via the AlphaFold2 and HDOCK algorithms suggests that this microneedle technology holds promise for clinical translation. Integration of meta-analyses of randomized controlled trials in tissue engineering [194] with spatiotemporal omics techniques to jointly evaluate efficacy may build a bridge between evidence-based medicine and mechanism elucidation. In addition, newly developed *in vivo* optical clearing agents (OCAs) [e.g. hyaluronic acid (HA)] rapidly increase skin transparency to enable 3D imaging at greater depths for precise ROI selection and longitudinal response monitoring. HA microneedles or HA-coated, charge-reversal nanomedicines (PPC@HA) can synergize with phototherapies to sensitize treatment-resistant port wine stains and cutaneous tumors [195, 196]. (iii) Mechanism elucidation: spatiotemporal omics enables the mechanistic dissection of how biomaterials modulate cellular fate decisions, CCC, immune activation states, and regenerative signaling cascades. This ability enables the selection of optimal mechanical, structural, or biochemical parameters, driving a transition from empirical material design to mechanism-driven optimization. In one study, scRNA-seq and ST were utilized to compare the reparative efficacy of ECM scaffolds in wound sites between young and aged mice [197]. The analysis revealed a specialized macrophage subpopulation, Mbm (Spp1^+^Arg1^+^), that accumulated in regions surrounding the scaffolds and served as a critical regulator of repair. Notably, aged Mbm macrophages lost the ability to activate Th2 immune responses but instead upregulated the immunosuppressive PD-L1 pathway, ultimately compromising reparative outcomes. These findings suggest that the design of biomaterials for use in elderly patients may necessitate combination with PD-L1 inhibitors or immunometabolic modulators to reverse age-related immunological inertia.

Across the four physiological and pathological contexts of development, aging, disease, and regeneration, the evolution of cellular heterogeneity and state transitions revealed by spatiotemporal omics follows fundamentally distinct organizational logics. During physiological development, heterogeneity is highly programmatic, with cell states progressing along relatively unidirectional, stagewise trajectories and spatial stratification and niche-derived cues providing precise coordinates for lineage commitment and fate specification. In contrast, aging is characterized by multipath trajectories of functional decline, in which cells progressively deviate from homeostasis and form inflammatory or senescence-associated hotspots in the spatial domain. These changes are often driven by local microenvironmental cues and retain a degree of reversibility. By comparison, cellular heterogeneity in pathological states is markedly dysregulated. Chronic inflammation, malignancy, and fibrosis do not simply reproduce or amplify physiological programs. Instead, they remodel CCC networks and metabolic states, stabilizing cells within new pathological attractor states and, in some cases, aberrantly reactivating development-associated programs. In contrast, wound reconstruction manifests a form of stress-induced, short-term plasticity in which multiple cell types undergo rapid state transitions over time and converge at specific spatial niches that critically determine whether outcomes are regenerative or fibrotic. On the basis of this integrative conceptualization, the core biological questions, major unresolved challenges, and skin-specific spatiotemporal omics gaps across the four domains are systematically summarized and compared in Table 2. This synthesis provides a reference framework for future cross-context comparative studies and mechanism-oriented investigations.

**Table 2:** Key questions in, challenges in, and translational relevance of skin spatiotemporal omics

Domain	Key questions	Major challenges	Skin-specific features	Translational relevance
Development	How are epidermal, appendage, mesenchymal, and immune lineages specified in time and space? How does epithelial–mesenchymal crosstalk shape appendage fate?	Limited access to human embryonic skin Incomplete conservation between humans and mice Sampling gaps at critical developmental time windows	Stratified epidermis with spatial signaling gradients Cyclic development of skin appendages Early immune involvement in tissue patterning	Reactivation of developmental pathways to target adult stem cells Guidance for stem-cell and organoid model establishment
Aging	Which cell states enter senescence earliest and at what sites? How does the senescence-associated secretory phenotype remodel the local microenvironment?	Loss of fragile senescent cells during dissociation Difficulty separating causal from correlative aging states	Regional clustering of senescent cells Strong coupling to hair follicle cycling Enhanced immune–stromal interactions	Identification of targetable aging niches Spatial stratification of aging phenotypes
Disease	How does cellular heterogeneity drive disease progression and treatment responses? How are cell–cell communication networks rewired?	Difficulty capturing early disease niches High intra- and interlesional heterogeneity Limited availability of longitudinal clinical samples	Clear spatial boundaries among lesional regions Reactivation of developmental programs extracellular matrix-driven spatial heterogeneity	Molecular stratification beyond histopathology Development of spatially informed immunotherapy
Regeneration	How do fibroblast, immune, and epithelial subpopulations determine repair outcomes? How do regenerative and fibrotic trajectories diverge in space and time? What are the precise mechanisms underlying wound-induced hair follicle neogenesis?	Conceptual overlap between repair and regeneration Limited ability to predict scarring outcomes	Fibroblast fate depends on depth and mechanics Spatially localized fibrotic hotspots	Guidance for antifibrotic therapy development and biomaterial design

Abbreviations: SASP, senescence-associated secretory phenotype; HF, hair follicle; ECM, extracellular matrix; WIHN, wound-induced hair follicle neogenesis

### Recent advances and future directions

#### Technological and algorithmic innovations

The application of spatiotemporal omics enables multidimensional analysis of tissue features, capturing both single-cell transcriptional heterogeneity and the local spatial architecture. However, its inherent complexity requires more efficient multimodal data analysis methods. In recent years, a series of novel technologies and algorithms have overcome bottlenecks in spatial domain identification, subcellular registration, and intercellular communication modeling, laying the foundation for constructing a true four-dimensional (4D) spatiotemporal skin atlas.

##### Spatial domain recognition

Accurate spatial domain identification is a prerequisite for characterizing the molecular architecture of skin [198, 199]. While traditional algorithms such as SpaGCN, BayesSpace, and stLearn [200] integrate scRNA-seq data with spatial data, they are often constrained by their linear modeling frameworks, limiting their performance in the analysis of highly heterogeneous skin tissues. In contrast, next-generation multimodal deep learning approaches, exemplified by DeepST [201], incorporate morphological features extracted from H&E images to improve boundary detection across complex skin layers and dermal–epidermal interfaces. Similarly, GNTD models spatial data as a space × gene × function tensor and applies graph-based nonlinear decomposition to address data sparsity in platforms such as Stereo-seq, enabling downstream spatial clustering and disease stratification [202]. In the latest algorithms, namely, TriCLEFF [203] and IRIS [198], robustness is further enhanced through the integration of contrastive learning and scRNA-seq references, which is particularly effective for the analysis of complex datasets such as those generated with the 10x Xenium platform and Stereo-seq. However, current ST platforms remain limited by their spot or pixel resolution, rendering spatial domain identification fundamentally a signal deconvolution problem [204, 205]. To overcome this constraint, imaging-based *in situ* transcriptomic technologies such as MERFISH [26] and seqFISH+ [206] have been developed to achieve near-single-molecule resolution, whereas ultrahigh-density array-based approaches such as Stereo-seq [34] support large-scale mapping at near-single-cell resolution. Furthermore, tools such as Spateo [207] extend spatial analysis toward dynamic modeling of cell–cell interactions, marking a transition toward integrated time–space–lineage frameworks in skin research.

##### Subcellular resolution

As spatial resolution advances toward the subcellular scale, the accurate assignment of transcripts to specific intracellular compartments is directly impacting downstream analyses. The UCS framework integrates high-density spatial expression matrices with histological images to achieve cross-platform subcellular alignment. By employing graph-based contrastive learning, UCS constructs RNA subcellular proximity networks, significantly increasing cell type annotation accuracy on the MERFISH and Xenium platforms. FOCUS, a complementary algorithm, has demonstrated particular utility for rare sample annotation where traditional models fail [208, 209]. Unlike traditional subcellular RNA purification methods, the PHOTON platform enables the analysis of membraneless structures and organelle-level transcripts without biochemical purification, offering a novel technique for subcellular omics [210].

##### Cell–cell communication modeling

Skin development, regeneration, and pathology rely on precise CCC. Traditional CCC inference methods (e.g. CellPhoneDB, NicheNet, and CellChat) rely on scRNA-seq–derived expression levels and ligand–receptor pairing but neglect the cellular spatial distribution and physical constraints. COMMOT addresses this limitation by incorporating a collective optimal transport model, integrating spatial positioning with signal diffusion pathway information to improve communication directionality and biophysical realism [211]. stMLnet models a four-tier (ligand, receptor, transcription factor, and target gene) feedback loop and can reveal hierarchical inter-tissue interactions, such as those involving epidermal–immune and fibroblast–KC signaling [212]. CrossChat employs cross-attention mechanisms to identify heterogeneous communication patterns across spatial locations, challenging the assumption that cell type is equivalent to communication behavior [213]. These methods offer novel insights for multiscale signal analysis and functional reconstruction.

##### *In vivo* dynamics

A crucial auxiliary technique for imaging-based spatial omics, skin OTC has transitioned from *ex vivo* to *in vivo* applications with the advent of OCAs. Absorbing molecules (e.g. tartrazine) increase the aqueous phase RI within minutes after topical application, resulting in reversible *in vivo* transparency at wavelengths higher than 600 nm, with the capability for millimeter-scale penetration and 3D imaging of mouse skin/muscle [214]. Biocompatible OCAs such as HA, delivered intradermally or transcutaneously, increase skin transparency within 30 min of application and promote collagen ordering, providing a safe, effective conduit for deeper *in vivo* observation and optical interventions. Consequently, imaging-based spatial omics methods (e.g. MERFISH and HCR-FISH) allow true volumetric construction of *in situ* molecular maps over larger tissue volumes in cleared skin and—after ROI/time point selection—can be coregistered with sequencing-based spatial omics approaches to obtain an integrated, dynamic spatiotemporal view of skin pathology. Representative studies utilizing skin OTC are summarized in Table 3.

**Table 3:** Representative studies applying optical tissue clearing in skin pathophysiology

Protocol/OCA	Pathophysiology	Model	Readout platform	Advantages	Ref.
CUBIC	Hair follicle development	*Ex vivo*, full-thickness mouse skin	Confocal microscopy	Enables true 3D, single-cell visualization of full-thickness skin across hair cycle stages (telogen/anagen), allowing quantitative mapping of follicles and adnexal structures	[77]
BABB	Psoriasis	*Ex vivo*, 5 mm full-thickness human skin	LSFM	Enables the visualization of psoriasiform lesion morphology—revealing marked epidermal thickening with focal parakeratosis, spongiotic changes with loss of the granular layer, and dense inflammatory infiltrates in the dermis	[112]
RapiClear 1.52	AD, psoriasis	*Ex vivo*, full-thickness human skin	Confocal microscopy	Provides standardized, 3D IENF quantification in the native microenvironment, revealing significant reductions in epidermal innervation in lesional AD and psoriasis	[119]
ACT-PRESTO	AD, psoriasis	*Ex vivo*, intact human epidermal sheets	Confocal microscopy	Provides whole-mount 3D reconstruction and standardized quantitative metrics of IENFs with improved linear integrity compared with the salt-split method	[121]
Skin-iDISCO+	Infantile hemangioma	*Ex vivo*, full-thickness human skin	Confocal microscopy, LSFM	Preserves adnexal matrix scaffolds with minimal distortion, enabling high-transparency 3D imaging and clearer delineation of tumor vascular boundaries	[146]
*In vivo* optical clearing skin window (OCSW)	Type 1 diabetes–related skin disorders	*In vivo*, dorsal skin of diabetic mice	TPLSM	Enables label-free, *in vivo* 3D quantification of dermal collagen changes and dynamic monitoring of cutaneous microvascular permeability	[160]
Modified BABB	Wound healing	*Ex vivo*, full-thickness mice skin	Confocal microscopy	Preserves endogenous fluorophores with minimal tissue shrinkage, enabling detailed 3D imaging of skin and wound healing biology	[173]
iDISCO	Keloid	*Ex vivo*, human keloid tissue samples	LSFM	Enables 3D visualization of keloid vasculature with quantitative assessment of vessel architecture	[174]
OCA (HA)	Tissue engineering	*In vivo*, dorsal skin of mice	Confocal microscopy, LSFM	HA with a high molecular weight and high concentration induces a lasting optical translucency effect alongside excellent biocompatibility, highlighting its potential for *in vivo* observation of skin dynamics	[195]
OCA (tartrazine)	Tissue engineering	*In vivo*, scalp and abdominal skin of mice	LSCI	Accomplishes reversible transparentization of the scalp and abdomen to enable repeatable, reversible *in vivo* imaging of cerebral vessels and internal organs	[214]

Abbreviations: *CUBIC* clear, unobstructed brain/body imaging cocktails and computational analysis, *BABB*, benzyl alcohol and benzyl benzoate, *TPLSM* two-photon laser-scanning microscopy, *iDISCO* immunolabeling-enabled three-dimensional imaging of solvent-cleared organs, *LSCI* laser speckle contrast imaging

##### Integrating the skin microbiome and spatiotemporal omics

The skin microbiome is considered essential for maintaining skin homeostasis and barrier function, whereas microbial dysbiosis has been linked to the onset and progression of many common skin diseases [215]. Through basic and clinical studies, researchers have increasingly recognized the pivotal role of the microbiota in wound healing, and this recognition may open new therapeutic avenues for patients with wounds [216]. The application of spatiotemporal omics is expanding beyond cellular- and tissue-level reconstruction to incorporate the skin microecosystem, enabling integrated analyses of host–microbiome spatial interactions. The construction of the integrated Human Skin Microbial Gene Catalog (iHSMGC), enabled by conventional metagenomic shotgun sequencing (BGISEQ-500 platform), together with the public Comprehensive Antibiotic Resistance Database (CARD), has allowed the characterization of the human skin resistome and revealed interethnic complexity within the skin microbiome [217]. The Unified Human Skin Genome (UHSG) catalog, covering a larger sample cohort, was assembled via ultradeep sequencing and integrated data analysis. Through multidimensional analyses, including single nucleotide variant, biosynthetic gene cluster, and horizontal gene transfer analyses, it prioritizes the novel annotation of microbial genomes, providing a convenient reference database for subsequent research [218].

In recent studies, metagenomics has been combined with ST to map microbe–host dynamics within skin niches. Notably, shotgun DNA sequencing, with its advantages of high coverage and resolution, can be used to identify the spatial clustering patterns of specific bacterial strains *in situ* [219]. By integrating these data with data obtained via spatial capture technologies, researchers have reconstructed microbe–host interaction networks associated with skin aging, revealing that certain bacterial consortia may drive phenotypic aging through metabolic reprogramming and inflammation-mediated pathways [220]. This multimodal framework is particularly promising for the study of skin diseases and pathologies involving microbiome dysbiosis, such as photoaging, eczema, and acne, where it has the potential to elucidate causal microbe–cell–microenvironment interaction chains. This insight offers a mechanistic foundation for personalized interventions. In the latest Stereo-seq V2 technology, random primers are employed to achieve the *in situ* capture of full transcripts, including nonpolyadenylated and microbial RNAs, from formalin-fixed, paraffin-embedded (FFPE) tissues [221]. This approach enables the spatially resolved analysis of host–microbe transcriptomic interactions at single-cell resolution and provides a more sensitive and broadly compatible platform for spatiotemporal omics studies of the skin microecosystem. Collectively, these emerging technologies and algorithms have significantly increased the ability to resolve multilayered, multiscale information within skin tissue. These advances are reshaping the systems-level understanding of skin development, physiology, and pathology, propelling the field toward the era of true 4D omics.

#### Challenges and perspectives

**Figure 5:**
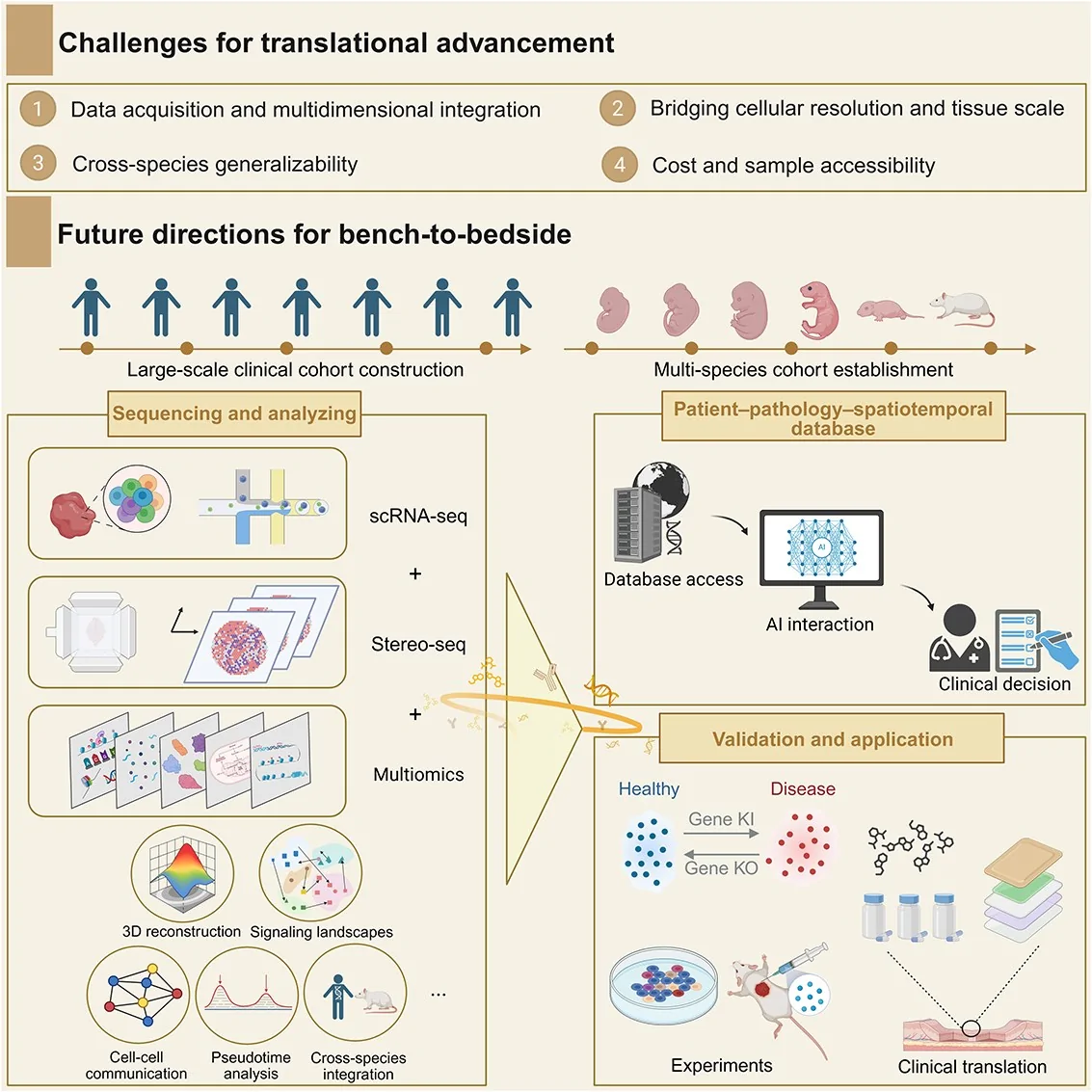
Challenges and future directions in spatiotemporal omics studies of skin biology. Upper section: four key challenges that hinder translational advancement. Lower section: future directions toward clinical translation. The establishment of large-scale clinical cohorts and multispecies cohorts provide the foundation for downstream multiomics integration. Left panel: Sequencing and analytical pipelines integrate scRNA-seq, stereo-seq, and multiomics profiling to enable 3D reconstruction, signaling landscape mapping, cell–cell communication analysis, pseudotime trajectory inference, and cross-species integration. Upper right: a patient–pathology–spatiotemporal database supports AI-assisted analysis and clinical decision-making. Bottom right: validation and application are achieved through functional experiments and clinical translation, linking fundamental discoveries to precision diagnostics and regenerative therapies. *KI* knock-in, *KO* knockout

Despite the breakthroughs in spatiotemporal omics for the mechanistic dissection of skin development, aging, disease, and regeneration, multiple challenges and limitations remain. Technological innovation and cross-disciplinary integration are urgently needed to advance the broader clinical translation of these approaches (Figure 5).

##### Skin-specific anatomical and physiological constraints

Compared to most solid organs, the skin possesses unique anatomical and physiological properties that present a series of organ-specific technical hurdles. One major obstacle lies in the realm of physical sample preparation and molecular penetration. The dense ECM of the dermis, particularly crosslinked collagen fibers, increases tissue rigidity and often leads to folding, tearing, and spatial distortion of tissue during sectioning. Furthermore, the ECM forms a natural permeability barrier that restricts reagent entry, necessitating optimized permeabilization parameters to balance RNA recovery with spatial diffusion [222]. Imaging-based spatial omics approaches are also limited by the substantial optical background interference in skin tissues [223]. Strong intrinsic autofluorescence and melanin-associated light absorption introduce significant background noise, resulting in increased demands for signal amplification, background suppression, and computational denoising strategies. Another challenge arises from sampling bias in 3D structures. Skin appendages such as HFs extend vertically through multiple layers, making it difficult for their complex spatial neighborhoods to be successfully reconstructed in conventional 2D sections. This limitation requires tissue clearing or serial section registration for integrated morphomolecular reconstruction. Finally, the marked differences in cell density and dissociation efficiency between the epidermis and dermis further complicate data interpretation. Fibroblasts and immune cells embedded within dense dermal matrices often have lower recovery rates, which potentially leads to distortion of cell type proportions in sequencing datasets. These factors underscore the necessity for a skin-specific spatiotemporal omics framework.

##### Standardized data processing and batch effect heterogeneity

As skin spatiotemporal omics methods have moved toward multisample and multicenter applications, the lack of unified data processing standards has become a key bottleneck for reproducibility. Differences in sample acquisition, analysis platforms, and annotation strategies often lead to inconsistent conclusions. While a universal pipeline has yet to be established, it is becoming increasingly feasible to develop standard operating procedures (SOPs) based on established toolkits such as Seurat [224], Scanpy [225], Squidpy [226], Giotto [227], and CellChat, which cover quality control, normalization, spatial denoising, and CCC analysis. In addition to this technical variance, the skin exhibits significant multilayered heterogeneity. Individual differences (age, sex, and disease state), anatomical site variations (sun-exposed *vs* non-sun-exposed and papillary *vs* reticular ratios), and technical discrepancies in sampling depth or fixation can significantly influence spatial signals. Batch effect correction must be addressed through both experimental design (randomized and balanced sampling) and computational frameworks (e.g. Harmony [228], LIGER [229], or Seurat integration). However, in skin tissues with distinct spatial layering, overcorrection risks masking authentic biological signals, necessitating cautious evaluation. Establishing standardized protocols spanning the entire workflow is fundamental to constructing high-fidelity spatiotemporal atlases of skin.

##### Cross-species generalizability

While mouse models are indispensable because of their standardizability, low cost, and numerous genetic toolkits, the evolutionary divergence between human and mouse skin presents a limitation for clinical translation. Anatomically, human skin features a thick epidermis and rete ridges, with healing achieved primarily via re-epithelialization and granulation. In contrast, mouse skin possesses a much thinner epidermis and a unique panniculus carnosus layer; thus, the wound closure process is dominated by mechanical contraction [230, 231]. These biomechanical differences profoundly influence the expression of mechanosensitive genes detected by spatial omics approaches. Humans also possess well-developed eccrine sweat glands, which are absent in mice, and exhibit marked differences in HF density and stem cell niche organization [232, 233]. From the molecular perspective, while the physiological stages of healing are similar [234, 235], single-cell data have revealed nonconserved lineage definitions. The binary lineage theory of mouse fibroblasts (e.g. the En1-defined lineage) lacks a validated equivalent in humans, where fibroblast organization is represented by a spatial continuum rather than a binary division [86, 167]. For instance, while CD39 is a marker for the papillary layer in both species, few mouse-defined reticular markers map conservatively to humans [15]. Immunologically, mice possess dendritic epidermal T cells (DETCs) that drive contraction, but these cells are absent in humans. Conversely, human skin harbors more complex tissue-resident Trm subsets and exhibits greater fibroblast heterogeneity [236]. Furthermore, mice exhibit robust WIHN, driven by Wnt/β-catenin signaling activation mediated by γδ T-cell–derived FGF9. In human wounds, this axis is suppressed, while TGF-β/Smad3 signaling predominates, promoting myofibroblast activation and fibrotic scarring [177, 178, 237]. These differences in pathway polarity help explain why regenerative therapies that are effective in mice often fail in clinical trials. Consequently, there is an urgent need to develop cross-species integration algorithms to bridge this evolutionary gap and to support their applicability by validation in humanized mice or skin-on-a-chip models.

##### Future perspectives

To overcome these barriers, the following directions should be prioritized in future efforts. First, in alignment with the Human Cell Atlas initiative [238], we propose the Spatial Chronological Omics of Pathophysiology in Cutaneous Ecosystems Plan (SCOPE): a nationwide, multidisciplinary effort to construct an integrative spatiotemporal framework of skin development, aging, disease, and regeneration. The aim of SCOPE is to assemble large-scale clinical and multispecies cohorts and to generate high-resolution multiomics resources for systematically charting the molecular and cellular state transitions underlying skin homeostasis and repair. These efforts would support the development of a scalable, publicly accessible online platform integrating multimodal single-cell and spatial omics data, thereby facilitating open data sharing and cross-institutional collaboration. At the mechanistic level, SCOPE is focused on the spatiotemporal dynamics of key cell populations and signaling pathways involved in wound healing and scar formation. From the translational perspective, we aim to establish original multicenter investigator-initiated trials (IITs) that connect mechanistic discoveries with clinical interventions, building an integrated ‘mechanism-to-clinic-to-evaluation’ research pipeline. Second, linking molecular insights to diagnostic and therapeutic practices should be prioritized in future work. For example, through ST on the 10x Genomics Visium platform, patients can be restratified on the basis of the molecular activity in local cellular ecosystems rather than reliance on conventional PASI scores alone. This approach enables the early detection of subclinical cellular activation and allows microscopic monitoring of long-term therapeutic efficacy. Moreover, spatiotemporal omics approaches enable comprehensive analysis of the highly heterogeneous TME, thereby increasing clinical efficacy. The spatial distribution, metabolic state, and CCC patterns of immune cells decisively influence the response to ICB therapy. A recent CosMx-based high-throughput spatiotemporal multiomics study revealed that PD-L1–high tumor cells in melanoma core regions colocalize with cells expressing key metabolic enzymes and are significantly correlated with nonresponse to immunotherapy. Through artificial intelligence (AI)-driven deep learning, algorithms such as DeepST and SpaSEG have demonstrated substantial potential in multidimensional data integration, spatiotemporal nested reconstruction, and predictive modeling. Looking ahead, the integration of high-throughput multimodal spatial omics techniques with AI is anticipated to facilitate the development of an open and interactive platform that integrates spatiotemporal atlas resources. Such a platform holds promise for equipping clinicians and researchers with intuitive tools for data exploration and evidence-based decision support.

## Conclusions

In this review, recent advances in and future directions for spatiotemporal omics in skin development, aging, disease, and regeneration are summarized. The integration of single-cell sequencing with spatial omics has demonstrated notable potential for evaluating cellular heterogeneity, spatial interactions, and dynamic changes. Emerging algorithms, cross-species strategies, and high-resolution spatial mapping enable more precise characterization of the skin microenvironment and construction of comprehensive atlases across the human lifespan. By leveraging AI and 4D modeling, spatiotemporal omics approaches aim to improve multidimensional data analysis, drive breakthroughs in personalized diagnostics and regenerative strategies, and accelerate clinical translation. Overall, these approaches are opening new avenues for precision dermatology and for a deeper mechanistic understanding of skin biology.

## Supplementary Material

Supplementary_Materials_tkag019
