# Supplementary material for: Spatiotemporal decoding of skin biology: development, aging, disease, and regeneration
Source: Burns Trauma. 2026 Feb 28;14:tkag019. doi: 10.1093/burnst/tkag019 (PMC13429052; doi:10.1093/burnst/tkag019)
Supplement: Supplementary_Materials_tkag019 [file supplementary_materials_tkag019.docx]

# **Supplementary Figures**


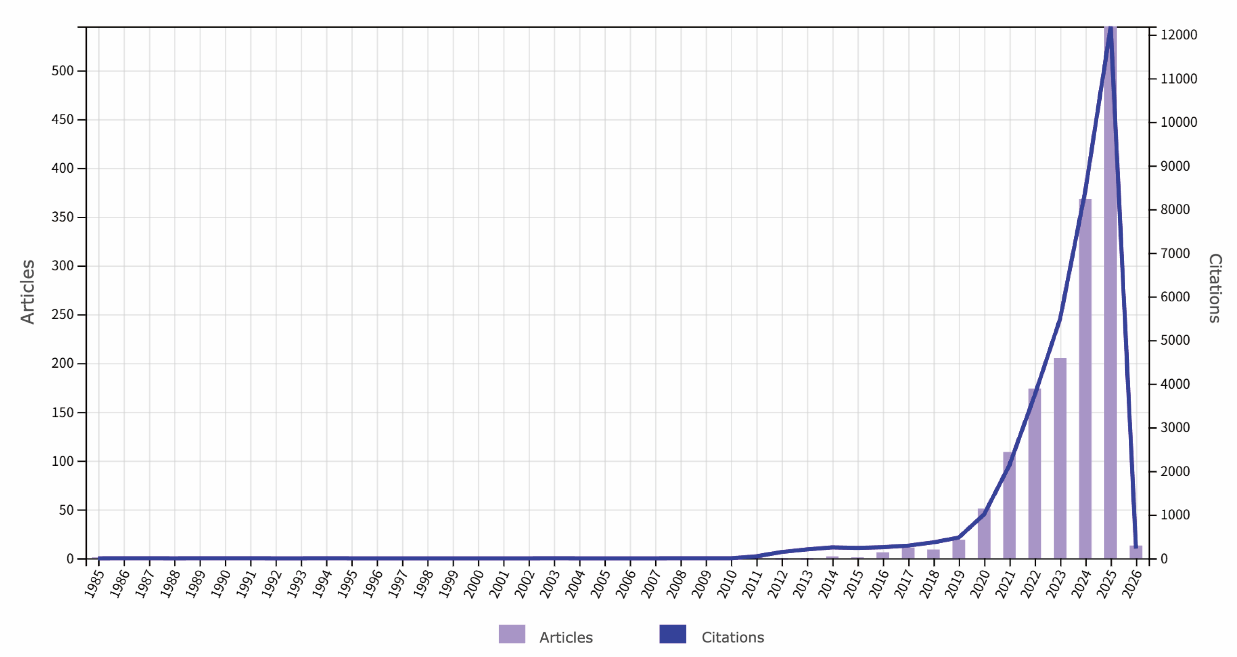


**Figure S1.** Quantity of annual scientific productions and citations.


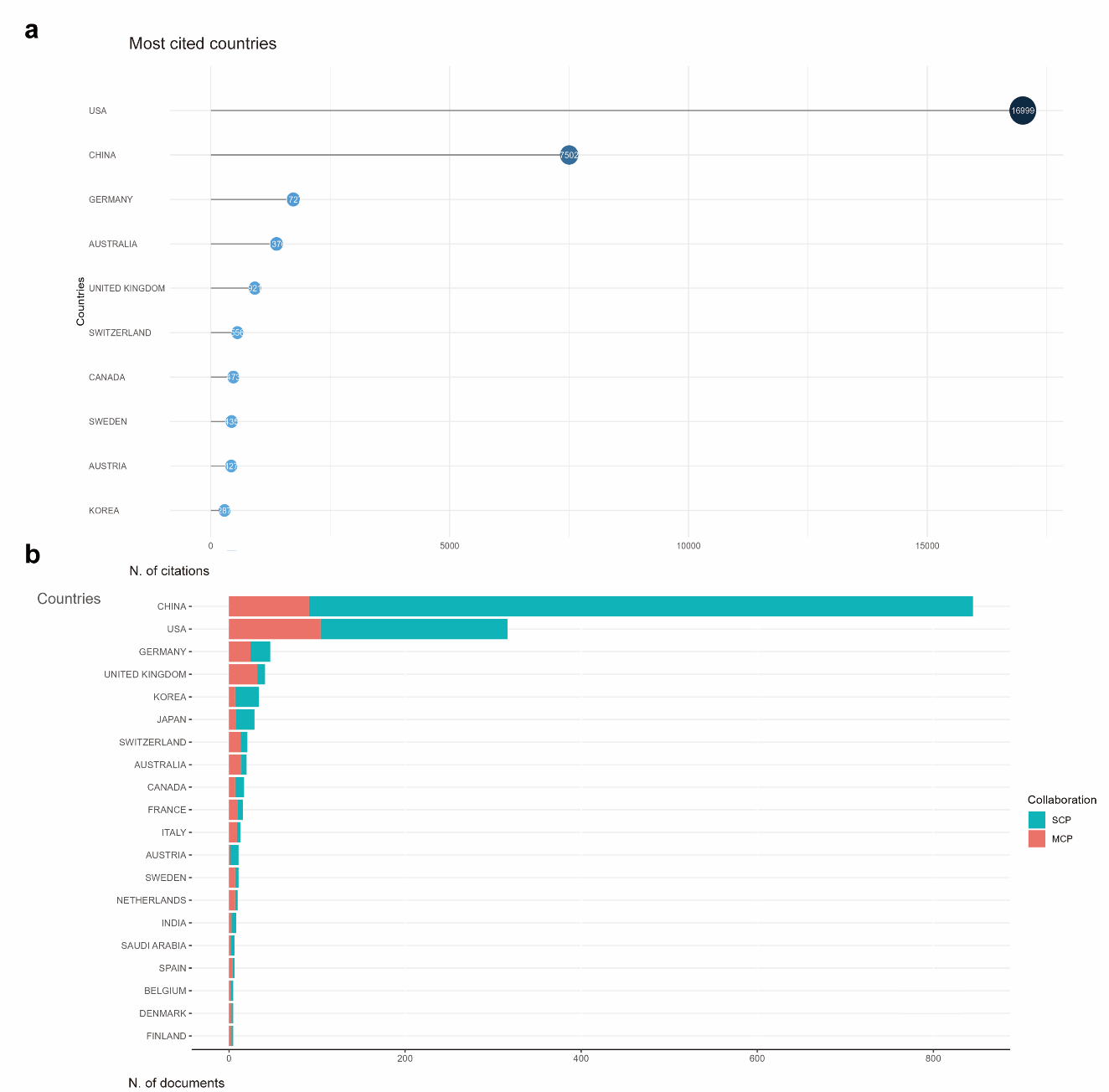


**Figure S2**. Supplementary materials for countries/regions production and collaboration analysis. (a) Top 10 most cited countries in skin spatiotemporal omics research. (b) SCPs (single-country publications) and MCPs (multiple-country publications) for the top 20 most productive countries.


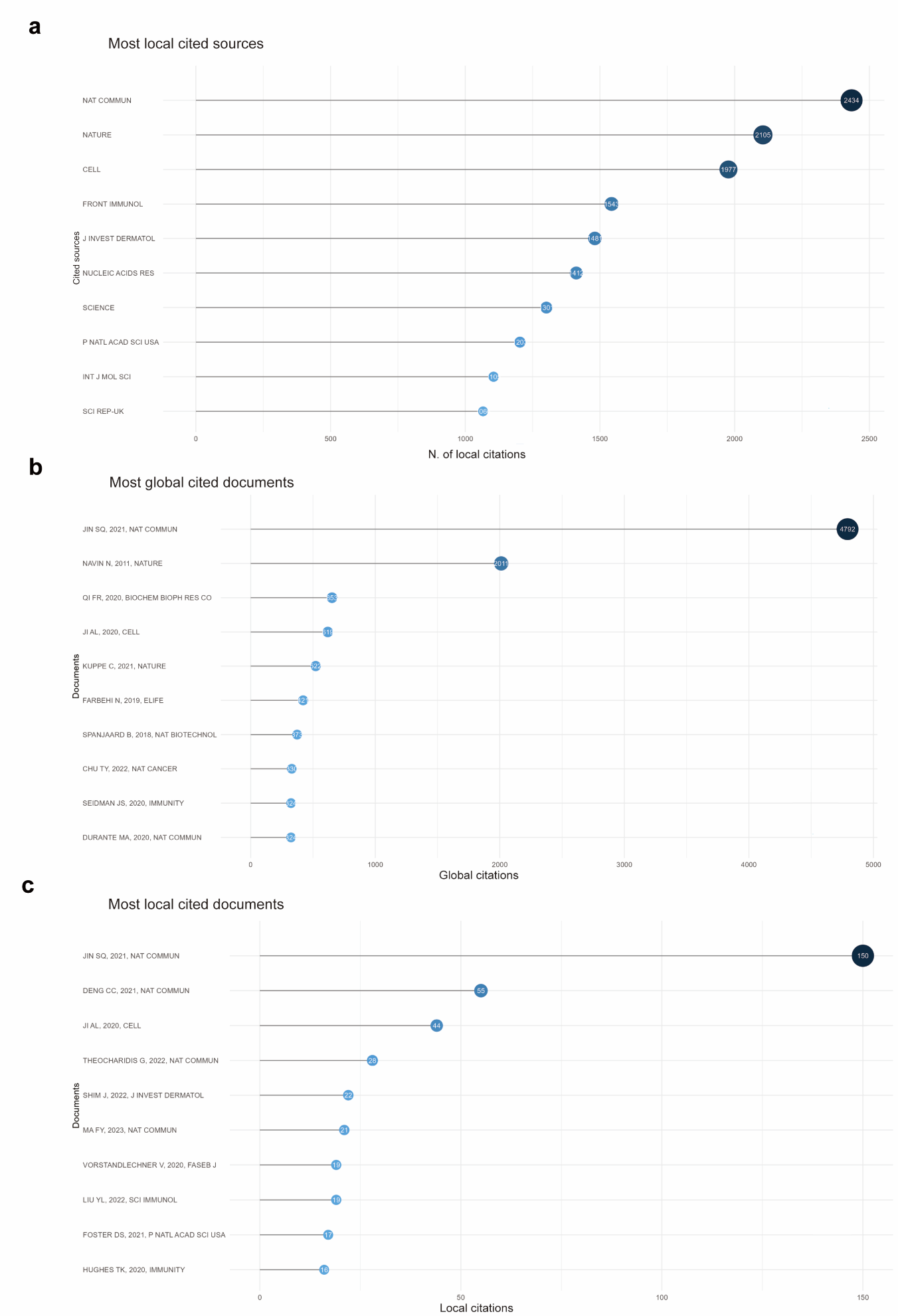


**Figure S3**. Supplementary materials for source and document analysis. (a) Top 20 most local cited journals in skin spatiotemporal omics research. (b) Top 20 most global cited documents. (c) Top 20 most local cited documents.


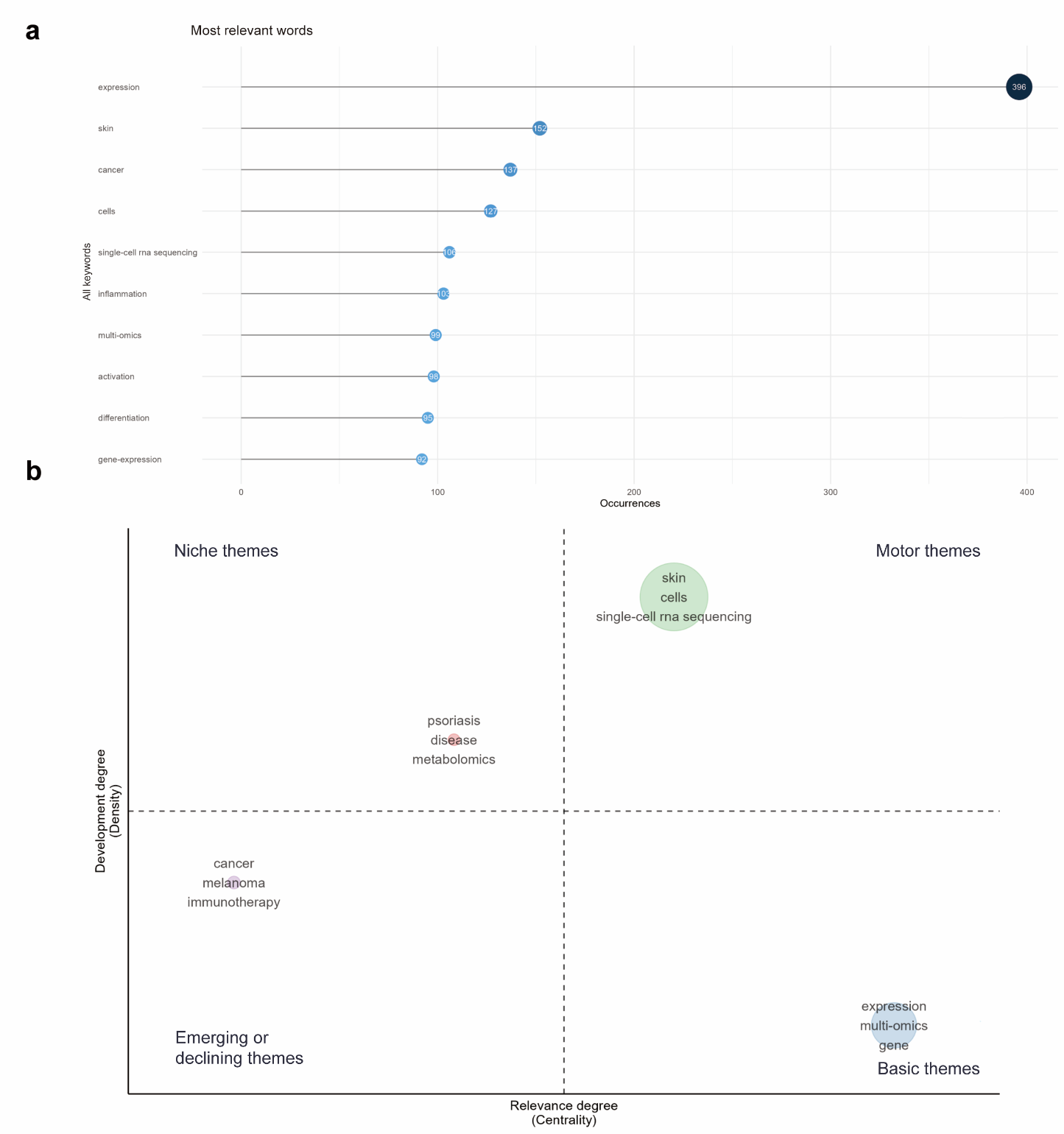


**Figure S4.** Supplementary materials for keyword and thematic analysis. (a) Top 10 most frequent keywords. (b) Thematic map of skin spatiotemporal omics research, constructed with centrality as the X-axis and density as the Y-axis. Each of the four quadrants points to the state of relevance and degree of development of different themes, which are respectively defined as motor themes, niche themes, emerging or declining themes, and basic themes.

# **Supplementary Tables**

| Table S1. Summary of existing algorithms for different tasks in spatiotemporal omics. | | | | | | |
| --- | --- | --- | --- | --- | --- | --- |
| **Type** | **Algorithm** | **Date** | **Functionality** | **Supported data types** | **Language** | **Ref** |
| Preprocessing | QuPath | 2017 | Digital pathology platform for whole-slide image analysis and annotation | H&E, IHC, fluorescence whole-slide images | Java | [1] |
|  | StarDist | 2018 | Deep-learning model for cell/nucleus segmentation with star-convex polygons | 2D/3D microscopy images | Python | [2] |
|  | Ilastik | 2019 | Interactive machine-learning platform for segmentation, classification, counting, and tracking | 2D/3D microscopy images | Python/C++ | [3] |
|  | Cellpose | 2021 | Generalised cell/nucleus partitioning | 2D/3D microscopy images | Python | [4] |
|  | Mesmer | 2022 | High-resolution spatial omics-based model for single-cell mask recognition | ST raw images (Stereo-seq, Xenium) | Python | [5] |
| Niche reconstruction and spatial partitioning | Harmony | 2019 | Cross-batch calibration and data integration | scRNA-seq | R | [6] |
|  | BBKNN | 2019 | Batch correction for scRNA-seq by constructing balanced kNN graphs across batches | scRNA-seq | Python | [7] |
|  | Stereoscope | 2020 | Probabilistic model for deconvoluting ST data using scRNA-seq reference profiles | scRNA-seq + ST | Python | [8] |
|  | BayesSpace | 2021 | Bayesian model for spatial domain detection and resolution enhancement | ST (10x Visium) | R | [9] |
|  | SpaGCN | 2021 | Graph convolutional network for integrating spatial coordinates, gene expression, and histology in spatial domain identification | ST (10x Visium, Slide-seq, Stereo-seq) | Python | [10] |
|  | Tangram | 2021 | Deep-learning model for alignment of scRNA-seq with ST | scRNA-seq + ST | Python | [11] |
|  | Cell2location | 2021 | Bayesian model for deconvolution of cell-type abundance in ST | scRNA-seq + ST | Python | [12] |
|  | Giotto | 2021 | Comprehensive toolkit for spatial omics analysis | ST, multiplex imaging | R/Python | [13] |
|  | STAGATE | 2022 | Graph attention autoencoder for spatial domain detection and denoising | ST (Visium, Slide-seq, Stereo-seq) | Python | [14] |
|  | DeepST | 2023 | Deep-learning framework for spatial domain identification and interaction analysis | ST (Visium, Slide-seq, Stereo-seq) | Python | [15] |
|  | SpaSEG | 2025 | GNN-based unsupervised spatial domain detection | ST (Visium, Slide-seq, Stereo-seq) | Python | [16] |
| Cell-cell communication analysis | NicheNet | 2019 | Ligand–target gene inference in intercellular communication | scRNA-seq + ST | R | [17] |
|  | CellPhoneDB 2.0 | 2020 | Ligand–receptor interaction inference | scRNA-seq | R/Python | [18] |
|  | CellChat | 2021 | CCC network inference and visualization | scRNA-seq + ST | R | [19] |
|  | imcRtools | 2022 | IMC data preprocessing and interaction analysis | IMC/ image-based ST | R | [20] |
|  | SpaTalk | 2022 | CCC detection in ST | scRNA-seq + ST | R | [21] |
|  | MISTy | 2022 | Multiview modeling of intercellular signaling | Spatial omics (IMC, MIBI, CODEX, Visium) | R | [22] |
|  | COMMOT | 2023 | Tool for directional CCC detection and communication inference | ST | Python | [23] |
| Trajectory inference | MARINa | 2009 | Master regulator inference | bulk/scRNA-seq | R/Java | [24] |
|  | ARACNe / ARACNe-AP | 2015 | GRN reconstruction | bulk/scRNA-seq | R/Java | [25] |
|  | DPT | 2016 | Diffusion pseudotime trajectory inference | scRNA-seq | R/Python | [26] |
|  | SCENIC | 2017 | GRN inference and TF activity scoring | scRNA-seq ± ST | R/Python | [27] |
|  | RNA velocity | 2018 | Future state prediction via splicing kinetics | scRNA-seq | Python | [28] |
|  | Monocle 3 | 2019 | Graph-based trajectory inference and pseudotime estimation | scRNA-seq | R | [29] |
|  | PAGA | 2019 | Graph abstraction for clustering and trajectory analysis | scRNA-seq | Python | [30] |
|  | cytoTRACE | 2020 | Cell differentiation potential and stemness scorring | scRNA-seq | R/Python | [31] |
|  | scVelo | 2020 | Dynamic RNA velocity analysis | scRNA-seq | Python | [32] |
|  | CellRank | 2021 | Model combining RNA velocity and Markov chain modeling for inferring cell fate probabilities and terminal states | scRNA-seq | Python | [33] |

Abbreviations: H&E, hematoxylin and eosin stain; IHC, immunohistochemistry; 2D/3D, two-dimensional/three-dimensional; ST, spatial transcriptomics; scRNA-seq, single-cell RNA sequencing; kNN, k-nearest neighbors; GNN, graph neural network; CCC, cell-cell communication; IMC, imaging mass cytometry; GRN, gene regulatory network; TF, transcription factor.

# **References**

1. Bankhead P, Loughrey MB, Fernández JA, Dombrowski Y, McArt DG, Dunne PD, et al. QuPath: Open source software for digital pathology image analysis. Sci Rep. 2017 Dec 4;7(1):16878.

2. Schmidt U, Weigert M, Broaddus C, Myers G. Cell Detection with Star-Convex Polygons. In: Frangi AF, Schnabel JA, Davatzikos C, Alberola-López C, Fichtinger G, editors. Medical Image Computing and Computer Assisted Intervention – MICCAI 2018. Cham: Springer International Publishing; 2018. p. 265–73.

3. Berg S, Kutra D, Kroeger T, Straehle CN, Kausler BX, Haubold C, et al. ilastik: interactive machine learning for (bio)image analysis. Nat Methods. 2019 Dec;16(12):1226–32.

4. Stringer C, Wang T, Michaelos M, Pachitariu M. Cellpose: a generalist algorithm for cellular segmentation. Nat Methods. 2021 Jan;18(1):100–6.

5. Greenwald NF, Miller G, Moen E, Kong A, Kagel A, Dougherty T, et al. Whole-cell segmentation of tissue images with human-level performance using large-scale data annotation and deep learning. Nat Biotechnol. 2022 Apr;40(4):555–65.

6. Korsunsky I, Millard N, Fan J, Slowikowski K, Zhang F, Wei K, et al. Fast, sensitive and accurate integration of single-cell data with Harmony. Nat Methods. 2019 Dec;16(12):1289–96.

7. Polański K, Young MD, Miao Z, Meyer KB, Teichmann SA, Park JE. BBKNN: fast batch alignment of single cell transcriptomes. Bioinformatics. 2020 Feb 1;36(3):964–5.

8. Andersson A, Bergenstråhle J, Asp M, Bergenstråhle L, Jurek A, Fernández Navarro J, et al. Single-cell and spatial transcriptomics enables probabilistic inference of cell type topography. Commun Biol. 2020 Oct 9;3(1):565.

9. Zhao E, Stone MR, Ren X, Guenthoer J, Smythe KS, Pulliam T, et al. Spatial transcriptomics at subspot resolution with BayesSpace. Nat Biotechnol. 2021 Nov;39(11):1375–84.

10. Hu J, Li X, Coleman K, Schroeder A, Ma N, Irwin DJ, et al. SpaGCN: Integrating gene expression, spatial location and histology to identify spatial domains and spatially variable genes by graph convolutional network. Nat Methods. 2021 Nov;18(11):1342–51.

11. Biancalani T, Scalia G, Buffoni L, Avasthi R, Lu Z, Sanger A, et al. Deep learning and alignment of spatially resolved single-cell transcriptomes with Tangram. Nat Methods. 2021 Nov;18(11):1352–62.

12. Kleshchevnikov V, Shmatko A, Dann E, Aivazidis A, King HW, Li T, et al. Cell2location maps fine-grained cell types in spatial transcriptomics. Nat Biotechnol. 2022 May;40(5):661–71.

13. Dries R, Zhu Q, Dong R, Eng CHL, Li H, Liu K, et al. Giotto: a toolbox for integrative analysis and visualization of spatial expression data. Genome Biol. 2021 Mar 8;22(1):78.

14. Dong K, Zhang S. Deciphering spatial domains from spatially resolved transcriptomics with an adaptive graph attention auto-encoder. Nat Commun. 2022 Apr 1;13(1):1739.

15. Xu C, Jin X, Wei S, Wang P, Luo M, Xu Z, et al. DeepST: identifying spatial domains in spatial transcriptomics by deep learning. Nucleic Acids Res. 2022 Dec 9;50(22):e131.

16. Bai Y, Guo X, Liu K, Zheng B, Wei Y, Wang Y, et al. SpaSEG: unsupervised deep learning for multi-task analysis of spatially resolved transcriptomics. Genome Biology. 2025 Jul 29;26(1):230.

17. Browaeys R, Saelens W, Saeys Y. NicheNet: modeling intercellular communication by linking ligands to target genes. Nat Methods. 2020 Feb;17(2):159–62.

18. Efremova M, Vento-Tormo M, Teichmann SA, Vento-Tormo R. CellPhoneDB: inferring cell-cell communication from combined expression of multi-subunit ligand-receptor complexes. Nat Protoc. 2020 Apr;15(4):1484–506.

19. Jin S, Guerrero-Juarez CF, Zhang L, Chang I, Ramos R, Kuan CH, et al. Inference and analysis of cell-cell communication using CellChat. Nat Commun. 2021 Feb 17;12(1):1088.

20. Windhager J, Zanotelli VRT, Schulz D, Meyer L, Daniel M, Bodenmiller B, et al. An end-to-end workflow for multiplexed image processing and analysis. Nat Protoc. 2023 Nov;18(11):3565–613.

21. Shao X, Li C, Yang H, Lu X, Liao J, Qian J, et al. Knowledge-graph-based cell-cell communication inference for spatially resolved transcriptomic data with SpaTalk. Nat Commun. 2022 Jul 30;13(1):4429.

22. Tanevski J, Flores ROR, Gabor A, Schapiro D, Saez-Rodriguez J. Explainable multiview framework for dissecting spatial relationships from highly multiplexed data. Genome Biol. 2022 Apr 14;23(1):97.

23. Cang Z, Zhao Y, Almet AA, Stabell A, Ramos R, Plikus MV, et al. Screening cell-cell communication in spatial transcriptomics via collective optimal transport. Nat Methods. 2023 Feb;20(2):218–28.

24. Lefebvre C, Rajbhandari P, Alvarez MJ, Bandaru P, Lim WK, Sato M, et al. A human B-cell interactome identifies MYB and FOXM1 as master regulators of proliferation in germinal centers. Mol Syst Biol. 2010 Jun 8;6:377.

25. Lachmann A, Giorgi FM, Lopez G, Califano A. ARACNe-AP: gene network reverse engineering through adaptive partitioning inference of mutual information. Bioinformatics. 2016 Jul 15;32(14):2233–5.

26. Haghverdi L, Büttner M, Wolf FA, Buettner F, Theis FJ. Diffusion pseudotime robustly reconstructs lineage branching. Nat Methods. 2016 Oct;13(10):845–8.

27. Aibar S, González-Blas CB, Moerman T, Huynh-Thu VA, Imrichova H, Hulselmans G, et al. SCENIC: single-cell regulatory network inference and clustering. Nat Methods. 2017 Nov;14(11):1083–6.

28. La Manno G, Soldatov R, Zeisel A, Braun E, Hochgerner H, Petukhov V, et al. RNA velocity of single cells. Nature. 2018 Aug;560(7719):494–8.

29. Cao J, Spielmann M, Qiu X, Huang X, Ibrahim DM, Hill AJ, et al. The single-cell transcriptional landscape of mammalian organogenesis. Nature. 2019 Feb;566(7745):496–502.

30. Wolf FA, Hamey FK, Plass M, Solana J, Dahlin JS, Göttgens B, et al. PAGA: graph abstraction reconciles clustering with trajectory inference through a topology preserving map of single cells. Genome Biol. 2019 Mar 19;20(1):59.

31. Gulati GS, Sikandar SS, Wesche DJ, Manjunath A, Bharadwaj A, Berger MJ, et al. Single-cell transcriptional diversity is a hallmark of developmental potential. Science. 2020 Jan 24;367(6476):405–11.

32. Bergen V, Lange M, Peidli S, Wolf FA, Theis FJ. Generalizing RNA velocity to transient cell states through dynamical modeling. Nat Biotechnol. 2020 Dec;38(12):1408–14.

33. Lange M, Bergen V, Klein M, Setty M, Reuter B, Bakhti M, et al. CellRank for directed single-cell fate mapping. Nat Methods. 2022 Feb;19(2):159–70.
